# Multimodal behavioral phenotyping for depressive-spectrum classification and severity estimation using eye tracking, facial behavior, and transcript-derived language

**DOI:** 10.3389/fpsyt.2026.1842005

**Published:** 2026-06-16

**Authors:** Xiang-Ting Chen, Min Huang

**Affiliations:** Department of General Medicine, The Affiliated Suzhou Hospital of Nanjing Medical University, Suzhou Municipal Hospital, Nanjing Medical University, Suzhou, Jiangsu, China

**Keywords:** eye tracking, facial behavior, major depressive disorder, multimodal machine learning, subthreshold depression

## Abstract

**Introduction:**

Depression assessment remains largely dependent on symptom reports and clinician judgment, while objective tools for depressive-spectrum stratification and severity estimation remain limited. Existing digital and multimodal depression-detection studies often focus on binary case-control classification, handle missing modalities incompletely, provide limited calibration assessment, and rarely combine depressive-spectrum classification with continuous symptom-severity estimation. We therefore developed a quality-aware multimodal framework integrating eye tracking, facial behavior, and transcript-derived language for classification across normal control (NC), subthreshold depression (SD), and major depressive disorder (MDD), together with prediction of 17-item Hamilton Depression Rating Scale (HAMD-17) severity.

**Methods:**

A total of 186 participants completed a controlled task battery including interview, emotional reading, free viewing with verbal description, fixation, gaze orienting, smooth pursuit, prosaccade, and antisaccade tasks. Eye-tracking, facial-video, and transcript-derived language data were converted into modality-specific features. Baseline-3 combined modality-specific encoders, quality-aware gated fusion, and joint classification-regression learning under a nested repeated-resampling framework with explicit missing-modality handling. Baseline-3+ further incorporated Transformer-based cross-modal interaction and uncertainty-based dynamic task weighting. Performance was evaluated on held-out outer-loop test sets after temperature scaling. Interpretability analyses included gate profiling, selective prediction, SHAP, Integrated Gradients, and counterfactual analysis.

**Results:**

Baseline-3+ showed the most favorable classification and calibration profile, with accuracy, balanced accuracy, and F1-macro approaching 0.90 across both classification routes and lower expected calibration error than Baseline-3. For severity estimation, the improvement was route-dependent and mainly reduced the regression disadvantage observed under the hierarchical route. Misclassification was concentrated near the SD boundary. Interpretability analyses showed stable quality-aware modality reweighting, with facial features providing the dominant signal, complemented by eye tracking and smaller but meaningful language contributions.

**Discussion:**

This framework addresses key limitations of prior binary and incompletely calibrated depression-detection models by jointly supporting depressive-spectrum classification, severity estimation, missing-modality handling, calibrated prediction, and individual-level interpretation. Its most plausible role is to augment clinical assessment, particularly for boundary states such as SD.

## 1 Introduction

Major depressive disorder (MDD) is a leading cause of disability worldwide and remains a major contributor to population disease burden (1). Despite its clinical importance, diagnosis still relies largely on symptom report and clinician judgment, and objective tools for screening, stratification, and longitudinal monitoring remain limited (2–6). Recent work in digital psychiatry and computational phenotyping has therefore focused on behavioral and physiological signals that may complement conventional assessment, although clinically transferable biomarkers for depression remain difficult to establish (7, 8). This challenge is especially relevant across the depressive spectrum. Subthreshold depression (SD) is more prevalent than syndromal MDD in many populations, is associated with functional impairment and elevated risk of progression, and is not adequately captured by symptom thresholds alone (9–12). Available evidence suggests that SD is not simply a milder form of MDD, but a heterogeneous intermediate state with variable symptom burden, psychosocial dysfunction, and transition risk. This heterogeneity also contributes to diagnostic ambiguity in data-driven models.

Behavioral markers used in depression-related modeling differ substantially in their biological and clinical meaning. Eye-tracking studies have linked depressive symptoms to altered visual attention, abnormal allocation to emotional stimuli, disturbed fixation, and impaired oculomotor control across fixation, saccade, and pursuit paradigms (13–19). These measures provide objective indices of attentional bias and cognitive control, although many studies remain focused on single-modality group discrimination or symptom association. Facial-behavior studies have used facial landmarks, head pose, and action-unit dynamics to quantify reduced expressivity, altered affective display, and impaired emotional flexibility (20–25). These features are clinically relevant because facial behavior forms part of routine mental-status assessment, but previous models have often emphasized binary depression detection or abnormalities in facial emotion processing rather than depressive-spectrum stratification. Language-based studies have examined transcript-derived text, acoustic speech, or audiovisual signals and have identified changes in fluency, self-reference, negation, affective wording, semantic structure, and speech characteristics (26–32). However, these studies differ in whether language is represented as text, audio, or combined speech-language information, and many focus on detecting depression status rather than jointly estimating clinical severity. Multimodal artificial intelligence has advanced automated depression assessment by integrating text, audio, facial behavior, eye tracking, and other behavioral signals (33–45). Existing models commonly use early fusion, late fusion, attention-based fusion, or deep representation learning to improve diagnostic discrimination, most often for binary depression detection or symptom-level prediction rather than simultaneous depressive-spectrum classification and severity estimation. These approaches have shown encouraging performance, but several limitations remain important for clinical translation. Many studies retain a binary case-control formulation, whereas fewer address intermediate depressive-spectrum states such as subthreshold depression. Missing or degraded modalities are often handled implicitly or by complete-case analysis, and probability calibration is rarely evaluated in detail. In addition, relatively few frameworks jointly model categorical depressive-spectrum status and continuous symptom severity while also providing individual-level interpretation. These gaps are particularly relevant when the target task extends beyond binary depression detection to spectrum-level stratification and symptom-severity estimation.

Against this background, we developed and evaluated a quality-aware multimodal framework that integrates eye tracking, facial behavior, and transcript-derived language acquired during a controlled behavioral task battery. The study was designed to move beyond conventional binary depression detection by modeling the depressive spectrum across normal control (NC), SD, and MDD, while also estimating continuous symptom severity using the 17-item Hamilton Depression Rating Scale (HAMD-17). Methodologically, the framework combines modality-specific representation learning, explicit modeling of modality availability and missingness, and quality-aware gated fusion, with an extended model incorporating Transformer-based cross-modal interaction and uncertainty-based dynamic task weighting. The evaluation further incorporated a nested repeated-resampling framework, *post hoc* probability calibration, component-ablation analyses, and individual-level interpretability. By linking spectrum-level classification, continuous severity estimation, missing-modality handling, calibrated prediction, and case-level explanation within a single framework, this study aimed to provide a more clinically interpretable approach for multimodal depressive-spectrum assessment, particularly for boundary states such as subthreshold depression. The framework was therefore evaluated as a calibrated and interpretable research prototype for depressive-spectrum stratification.

## 2 Materials and methods

### 2.1 Participants

Participants were recruited from Suzhou Municipal Hospital and Suyuan Community Health Service Station in Wuzhong District, Suzhou, China. The cohort comprised normal control (NC), subthreshold depression (SD), and major depressive disorder (MDD) groups. Diagnostic assessment was performed by licensed psychiatrists. MDD was diagnosed according to the International Classification of Diseases, 11th Revision (ICD-11), whereas SD and NC were defined using prespecified operational criteria supported by standardized rating scales. Clinical assessment and multimodal data acquisition were completed on the same day. The study was approved by the Ethics Committee of Suzhou Municipal Hospital (K-2025-278-K01). Written informed consent was obtained from all participants before enrollment. Detailed eligibility criteria and operational group definitions are provided in Appendix A.

### 2.2 Experimental procedures

All assessments were conducted in a controlled laboratory setting. Participants were seated in a quiet room, instructed to maintain a natural posture, and asked to minimize large head movements. Eye movements were recorded using a Tobii Pro Nano eye tracker at 60 Hz, mounted below a 14.5-inch laptop display (1920 × 1080 pixels), with a viewing distance of approximately 60–70 cm. Facial behavior was recorded using an external high-definition camera at 1080p and 30 frames/s. Spoken responses were transcribed for downstream language-feature extraction and data-quality control; acoustic speech features were not modeled as an independent modality. Before formal testing, a nine-point eye-tracking calibration was completed and repeated if necessary. Standardized instructions were provided before each task, and brief practice trials were given when required. A 30-s rest interval was arranged between adjacent tasks. The task battery included a sociodemographic questionnaire, a semi-structured interview, emotional text reading, emotional free viewing with verbal description, fixation stability, lateral gaze orienting, smooth pursuit, prosaccade, and antisaccade paradigms. Horizontal and sinusoidal pursuit conditions were both included. The interview and free-viewing tasks yielded transcript-derived language, whereas emotional text reading contributed eye-tracking and facial-behavior responses but was not used as an independent source for language modeling. Detailed task procedures are provided in Appendix B.

### 2.3 Multimodal preprocessing and feature engineering

Raw eye-tracking signals were processed using the velocity-threshold identification algorithm to classify fixations, saccades, and blinks. Samples outside the display region were discarded, and trials with a tracking ratio below 50% were excluded. A total of 723 participant-level eye-tracking features were extracted, covering fixation, visit, gaze transition, saccadic control, and higher-order cognitive and affective indices. Facial videos were analyzed frame by frame using OpenFace 2.0 to derive facial landmarks, head-pose measures, and facial action unit features. Frames with confidence below 0.7 or failed tracking were removed, and segments with a valid-frame ratio below 50% were excluded. Summary statistics and composite indices were then generated to quantify facial movement, affective expression, and task-related emotional regulation. The language modality was derived from offline transcription of spoken responses using Whisper large-v3 deployed through an OpenVINO-based workflow. Text preprocessing included normalization, sentence segmentation, identification of short segments, and conservative token filtering. Features were computed at the segment level and aggregated at the participant level within each task and emotional condition. The final language feature space included completeness and quality measures, structural and fluency indices, clinically relevant linguistic markers, and affective lexical features.

Across modalities, preprocessing, feature filtering, standardization, and feature selection were performed strictly within the training data of each resampling split to prevent information leakage. In the present study, modalities were defined by distinct data representations and processing pipelines, namely oculomotor behavior, facial behavioral dynamics, and transcript-derived linguistic features, rather than by whether they originated from separate acquisition sessions. Further details are provided in Appendix C. The outer-test data were transformed using parameters and feature sets learned exclusively from the corresponding training partition. To improve reproducibility and provide an auditable basis for subsequent modeling, the three modality-specific participant-level feature tables were first harmonized into a single raw multimodal master table before resampling-based model development. Eye-tracking, facial-behavior, and transcript-derived language features were prefixed by modality and aligned using a unique participant identifier. Diagnostic group labels and HAMD-17 scores were cross-checked across the three source tables before consensus labels were assigned. No imputation, winsorization, standardization, feature filtering, feature selection, calibration, or model training was performed during master-table construction. These procedures were deliberately deferred to the training data within each resampling split to prevent information leakage. The master table also retained modality-availability indicators and raw modality-specific missingness ratios for subsequent missing-modality modeling. Additional details on data alignment, master-table construction, missing-modality handling, and resampling-based model development are provided in Appendix D.

### 2.4 Multimodal modeling

Tabular features from the eye-tracking, facial, and language modalities were aligned by unique participant identifier. The classification target was depressive-status group membership (NC, SD, or MDD), and the regression target was HAMD-17. To accommodate incomplete multimodal acquisition, modality-level meta-features were constructed, including availability indicators and modality-specific missing ratios. Entire feature blocks for unavailable modalities were set to zero after fold-specific preprocessing. Model development followed a nested repeated-resampling framework. The three diagnostic groups were balanced by design, with 62 participants in each group. In the outer loop, data were repeatedly divided into training and held-out test sets using stratified 85:15 random partitioning over five repeats. Within each outer-training partition, five-fold stratified inner cross-validation was used for hyperparameter selection and early stopping. This design preserved class distribution across training and test partitions. In addition to ACC, we reported BACC, F1-macro, and class-level recall to avoid overinterpreting overall accuracy. Within each inner-loop training fold, modality-specific preprocessing included missingness filtering, median imputation, winsorization, low-variance filtering, redundancy reduction based on Spearman correlation, and z-score normalization. Supervised feature selection was then performed using repeated subsampling stability selection with elastic-net multinomial logistic regression. Because the downstream framework jointly modeled depressive-spectrum classification and HAMD-17 regression, feature selection was anchored to the classification structure to retain stable disease-relevant signals, while continuous severity estimation was learned jointly within the subsequent multimodal model.

The primary multimodal model, Baseline-3, combined modality-specific multilayer perceptron encoders with quality-aware gated fusion and joint classification-regression learning. For each modality *m*, where m∈{eye, face,  text}, the preprocessed input vector *x_m_* was mapped to a latent modality representation by a modality-specific encoder, $h_{m}=f_{m}\left(x_{m}\right)$. Quality metadata *q_m_*, including modality-availability indicators and modality-specific missingness ratios, were used together with modality embeddings to generate normalized gating weights *α_m_*. The Baseline-3 fused representation was then defined as $h=\underset{m}{\sum }\alpha_{m}h_{m}$, and was passed to separate classification and HAMD-17 regression heads. Baseline-3+ extended this architecture by treating modality embeddings as modality tokens and processing them through a lightweight Transformer encoder to obtain an interaction-aware shared representation. The multitask objective jointly optimized depressive-spectrum classification and HAMD-17 regression. In Baseline-3+, uncertainty-based dynamic task weighting assigned learnable task-uncertainty parameters to the classification and regression losses, allowing the relative contribution of each task to be adjusted during optimization. PCGrad was not included in the full Baseline-3+ model and was evaluated separately as a sensitivity arm under fixed task weighting. The full mathematical formulations of modality-specific encoding, quality-aware gated fusion, Route A and Route B probability reconstruction, Transformer-based interaction, dynamic task weighting, temperature scaling, expected calibration error, and ablation settings are provided in **Supplementary Appendix E**.

**Figure 1:**
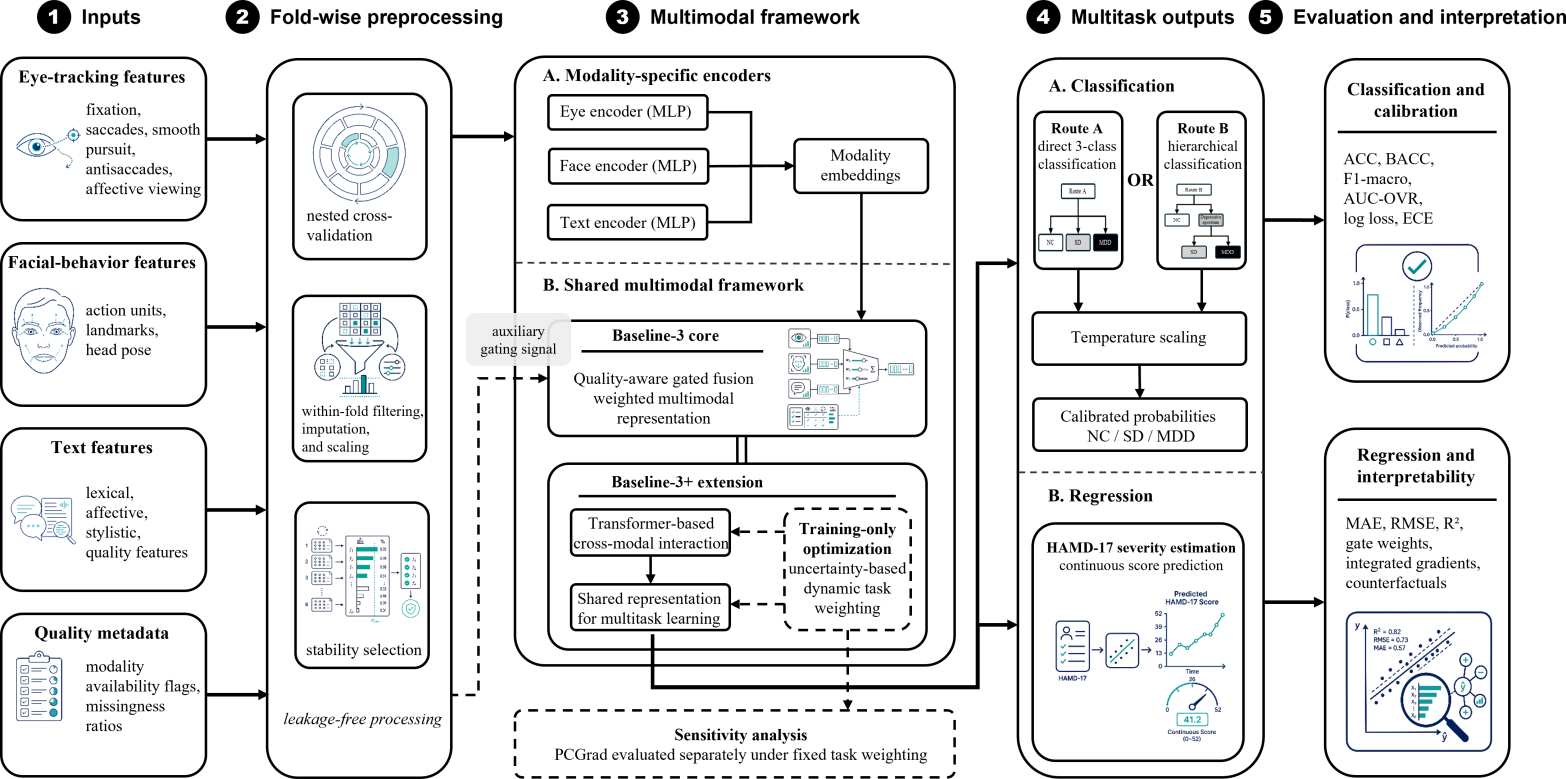
Overview of the quality-aware multimodal framework for depressive-spectrum classification and severity estimation. The image summarizes the overall study workflow. Multimodal inputs included eye-tracking, facial-behavior, and transcript-derived language features together with quality metadata describing modality availability and missingness. After resampling-specific preprocessing and feature selection, modality-specific multilayer perceptron encoders generated modality embeddings. In Baseline-3, these embeddings were integrated by quality-aware gated fusion, whereas Baseline-3+ further incorporated Transformer-based cross-modal interaction and uncertainty-based dynamic task weighting; PCGrad was evaluated separately as a sensitivity analysis under fixed task weighting. The shared representation supported multitask outputs for depressive-spectrum classification and HAMD-17 severity estimation, followed by calibration and interpretability analyses. ACC, accuracy; AUC-OVR, one-vs-rest macro-averaged area under the receiver operating characteristic curve; BACC, balanced accuracy; ECE, expected calibration error; HAMD-17, 17-item Hamilton Depression Rating Scale; MDD, major depressive disorder; MLP, multilayer perceptron; NC, normal control; PCGrad, Projected Conflicting Gradient; SD, subthreshold depression.

Two classification routes were implemented. Route A directly predicted NC, SD, and MDD. Route B used a hierarchical two-stage design that first separated NC from the depressive spectrum and then distinguished SD from MDD within spectrum-positive samples. Training incorporated modality dropout, AdamW optimization, and gradient clipping. Modality dropout was applied only during training; during each training iteration, available modality embeddings could be randomly masked according to the hyperparameter selected within the inner cross-validation loop. Validation and outer-test evaluation used the true observed modality-availability pattern without artificial dropout. Temperature-scaling parameters were fitted on validation predictions derived exclusively from the training portion of each outer split and were then applied unchanged to the corresponding outer-test predictions. The overall architecture of the proposed quality-aware multimodal framework, including fold-wise preprocessing, modality-specific encoding, quality-aware gated fusion, Baseline-3+ extensions, multitask outputs, calibration, and interpretability analysis, is summarized in **Figure 1**.

### 2.5 Evaluation and interpretability

Classification performance was assessed using accuracy (ACC), balanced accuracy (BACC), one-vs-rest macro-averaged area under the receiver operating characteristic curve (AUC-OVR), macro-averaged F1 score (F1-macro), and log loss. Regression performance was assessed using mean absolute error (MAE), root mean squared error (RMSE), and coefficient of determination (R^2^). Metrics were calculated on the outer-loop test sets and summarized across repeated splits as mean ± standard deviation. Interpretability was evaluated at both the modality and individual levels. Gating-weight distributions were examined overall, by diagnostic group, and by missingness burden. Individual-level explanations were derived using Integrated Gradients (IG) and counterfactual analysis under modality-availability constraints. To characterize factors associated with classification failure and regression error, surrogate models based on uncertainty, calibration, missingness, gating, and attention features were additionally analyzed using SHapley Additive exPlanations (SHAP). Full details of the fusion models, calibration, evaluation metrics, and interpretability analyses are provided in **Supplementary Appendix E**. Case-level interpretability analyses were conducted on the held-out outer-test set of one representative Baseline-3+ Route B outer repeat selected for illustrative analysis. This test set comprised 28 participants. To further address calibration and statistical robustness, we re-analyzed the original outer-test prediction files after temperature scaling. Expected calibration error was calculated using the maximum calibrated class probability and empirical correctness across 10 equal-width confidence bins. Metric values were summarized across the five outer-loop test repeats as mean with 95% confidence intervals. Ninety-five percent confidence intervals were calculated across the five outer repeats using the t distribution and were interpreted as descriptive resampling uncertainty summaries rather than as population-level inferential intervals. Paired comparisons between Baseline-3 and Baseline-3+ were performed across matched outer repeats using sign-flip permutation tests. Because only five outer repeats were available, these tests were interpreted as exploratory repeat-consistency analyses rather than confirmatory hypothesis tests.

Component-level ablation analyses were conducted to clarify the contribution of the main Baseline-3+ extensions. The full Baseline-3 and full Baseline-3+ models were retained as reference settings. Additional ablation settings selectively removed uncertainty-based dynamic task weighting or Transformer-based cross-modal interaction, and a PCGrad sensitivity arm was evaluated under fixed task weighting. Ablation results were summarized using the same outer-test discrimination, calibration, and HAMD-17 regression metrics as the full models. To provide a representative conventional reference aligned with common early-fusion tabular modeling strategies, we additionally evaluated regularized linear benchmark models within the same nested repeated-resampling framework. Elastic-net logistic regression was used for depressive-spectrum classification, and Ridge regression was used for HAMD-17 severity estimation. These models were selected *a priori* because they are interpretable and appropriate for high-dimensional small-sample tabular data. The benchmark analyses were performed using stability-selected single-modality features and early-fusion features across all modalities. These conventional models were interpreted as reference analyses of the extracted feature space rather than as replacements for the proposed quality-aware multitask framework, because they do not model modality quality, joint classification-regression learning, calibrated uncertainty, or individual-level multimodal explanation.

## 3 Results

### 3.1 Participant characteristics

A total of 186 participants were included, comprising 62 NC, 62 individuals with SD, and 62 patients with MDD. The reproducible multimodal master table contained 723 eye-tracking features, 2021 facial-behavior features, and 1029 transcript-derived language features. Cross-table verification identified no inconsistencies in diagnosis labels or HAMD-17 scores. Complete three-modality data were available for 168 participants, whereas 18 participants had at least one unavailable modality, supporting the use of modality-availability encoding and missingness-aware fusion. The master-table construction and modality-availability patterns are summarized in **Supplementary Table 1** and **S2**. Baseline demographic and clinical characteristics are presented in **Table 1**. The three groups were broadly comparable in age, sex, education, marital status, offspring status, living arrangement, and eye-tracking acquisition quality. Occupational distribution and body mass index showed significant omnibus differences, although no adjusted pairwise comparison remained significant after multiple-testing correction. Several lifestyle and psychosocial variables differed across groups. Satisfaction with current income, sleep status, dietary pattern, physical exercise, cognitive activity, social participation, and psychological disclosure all showed significant between-group differences, generally shifting from more favorable profiles in NC toward less favorable profiles in SD and MDD. By contrast, smoking history, alcohol use, and household-work involvement did not differ significantly. Clinical symptom burden also differed markedly. Scores on the Center for Epidemiologic Studies Depression Scale, the 17-item Hamilton Depression Rating Scale (HAMD-17), the 7-item Generalized Anxiety Disorder scale, and the 14-item Hamilton Anxiety Rating Scale increased stepwise from NC to SD to MDD, with significant pairwise differences throughout. Anxiety-category distribution showed the same gradient. Together, these findings were consistent with the expected clinical ordering of the three groups and indicated increasing depressive and anxiety burden across the spectrum.

**Table 1:** Demographic and clinical characteristics of the study groups (N = 186).

Characteristic	NC (n=62)	SD (n=62)	MDD (n=62)	χ²/H	*P* value
Age, y	54.50 (31.25, 65.00)	44.00 (30.00, 59.75)	40.50 (29.25, 56.75)	5.826	0.054
Sex, n (%)				3.150	0.207
Female	39 (62.90%)	44 (70.97%)	48 (77.42%)		
Male	23 (37.10%)	18 (29.03%)	14 (22.58%)		
Education level, n (%)				7.153	0.520
Compulsory education	17 (27.42%)	12 (19.35%)	12 (19.35%)		
Basic education	8 (12.90%)	14 (22.58%)	16 (25.81%)		
Vocational education	1 (1.61%)	4 (6.45%)	3 (4.84%)		
Professional education	31 (50.00%)	26 (41.94%)	28 (45.16%)		
Postgraduate education	5 (8.06%)	6 (9.68%)	3 (4.84%)		
Employment type, n (%)				14.685	0.023 ^d^
Mainly manual labor	9 (14.52%)	13 (20.97%)	11 (17.74%)		
Mixed manual and mental labor	15 (24.19%)	18 (29.03%)	17 (27.42%)		
Mainly mental labor	35 (56.45%)	30 (48.39%)	23 (37.10%)		
Unemployed	3 (4.84%)	1 (1.61%)	11 (17.74%)		
Satisfaction with current income				28.032	< 0.001 a^,b,c^
Dissatisfied	6 (9.68%)	8 (12.90%)	22 (35.48%)		
Neutral	27 (43.55%)	41 (66.13%)	30 (48.39%)		
Satisfied	29 (46.77%)	13 (20.97%)	10 (16.13%)		
Marital status, n (%)				9.582	0.143
Married	45 (72.58%)	42 (67.74%)	38 (61.29%)		
Unmarried	13 (20.97%)	19 (30.65%)	18 (29.03%)		
Divorced	0 (0%)	0 (0%)	3 (4.84%)		
Widowed	4 (6.45%)	1 (1.61%)	3 (4.84%)		
Children, n (%)				3.708	0.157
With children	47 (75.81%)	41 (66.13%)	37 (59.68%)		
Without children	15 (24.19%)	21 (33.87%)	25 (40.32%)		
Living arrangement, n (%)				1.447	0.485
Living alone	11 (17.74%)	15 (24.19%)	10 (16.13%)		
Not living alone	51 (82.26%)	47 (75.81%)	52 (83.87%)		
Height, m	1.63 (1.58, 1.70)	1.62 (1.60, 1.68)	1.60 (1.60, 1.66)	0.448	0.799
Weight, kg	58.15 (53.62, 67.00)	55.00 (52.00, 64.75)	54.00 (50.00, 64.50)	5.527	0.063
BMI, kg/m²	22.75 (20.58, 24.23)	21.61 (19.84, 22.94)	20.89 (19.56, 23.07)	6.605	0.037 ^d^
Sleep status, n (%)				75.912	< 0.001 a^,b,c^
Normal	39 (62.90%)	14 (22.58%)	4 (6.45%)		
Poor sleep quality	21 (33.87%)	27 (43.55%)	15 (24.19%)		
Insomnia	2 (3.23%)	21 (33.87%)	43 (69.35%)		
Dietary pattern, n (%)				26.763	< 0.001 ^b,c^
Mainly vegetarian	4 (6.45%)	2 (3.23%)	20 (32.26%)		
Balanced	52 (83.87%)	56 (90.32%)	39 (62.90%)		
Mainly meat-based	6 (9.68%)	4 (6.45%)	3 (4.84%)		
Smoking status, n (%)				3.015	0.807
Never	53 (85.48%)	53 (85.48%)	50 (80.65%)		
Occasional	2 (3.23%)	3 (4.84%)	5 (8.06%)		
Regular	3 (4.84%)	4 (6.45%)	5 (8.06%)		
Quit	4 (6.45%)	2 (3.23%)	2 (3.23%)		
Alcohol use, n (%)				7.089	0.313
Never	46 (74.19%)	49 (79.03%)	38 (61.29%)		
Occasional	12 (19.35%)	9 (14.52%)	18 (29.03%)		
Regular	2 (3.23%)	2 (3.23%)	5 (8.06%)		
Quit	2 (3.23%)	2 (3.23%)	1 (1.61%)		
Physical exercise, n (%)				37.366	< 0.001 a^,b,c^
Never	13 (20.97%)	29 (46.77%)	45 (72.58%)		
Occasional	22 (35.48%)	21 (33.87%)	11 (17.74%)		
Regular	27 (43.55%)	12 (19.35%)	6 (9.68%)		
Cognitive activities, n (%)				19.350	< 0.001 ^c^
Never	23 (37.10%)	37 (59.68%)	47 (75.81%)		
Occasional	25 (40.32%)	15 (24.19%)	9 (14.52%)		
Regular	14 (22.58%)	10 (16.13%)	6 (9.68%)		
Housework, n (%)				8.298	0.081
None	2 (3.23%)	9 (14.52%)	12 (19.35%)		
Partial	36 (58.06%)	35 (56.45%)	31 (50.00%)		
All	24 (38.71%)	18 (29.03%)	19 (30.65%)		
Social activities, n (%)				66.599	< 0.001 a^,b,c^
None	5 (8.06%)	33 (53.23%)	49 (79.03%)		
1–3/month	41 (66.13%)	21 (33.87%)	11 (17.74%)		
4–6/month	9 (14.52%)	3 (4.84%)	0 (0%)		
≥6/month	7 (11.29%)	5 (8.06%)	2 (3.23%)		
Emotional disclosure, n (%)				70.460	< 0.001 a^,b,c^
None	4 (6.45%)	32 (51.61%)	50 (80.65%)		
Occasional	39 (62.90%)	21 (33.87%)	10 (16.13%)		
Frequent	19 (30.65%)	9 (14.52%)	2 (3.23%)		
Tracking accuracy	0.67 (0.54, 0.95)	0.78 (0.54, 1.07)	0.69 (0.48, 1.18)	2.302	0.316
Tracking precision	0.49 (0.28, 0.90)	0.74 (0.45, 1.35)	0.69 (0.35, 1.24)	5.396	0.067
CES-D score	4.00 (2.00, 8.75)	13.00 (10.00, 17.00)	20.50 (18.00, 26.00)	107.361	< 0.001 a^,b,c^
HAMD-17 total score	2.00 (1.00, 3.00)	10.00 (9.00, 12.00)	19.00 (18.00, 21.00)	165.160	< 0.001 a^,b,c^
GAD-7 score	2.00 (0.00, 3.00)	9.00 (6.00, 12.00)	12.00 (8.00, 15.00)	101.199	< 0.001 a^,b,c^
HAMA-14 score	2.00 (1.00, 3.00)	9.00 (5.25, 11.75)	13.50 (9.00, 16.00)	105.296	< 0.001 a^,b,c^

Continuous variables are presented as median with the 25th and 75th percentiles because none met the assumptions of normality and homogeneity of variance. Between-group comparisons for continuous variables were performed using the Kruskal–Wallis test, followed by Dunn *post hoc* testing with Bonferroni correction. Categorical variables are presented as n percent and were compared using the chi-square test or Fisher exact test, as appropriate. Superscripts indicate significant pairwise differences after correction for multiple comparisons. a indicates NC versus SD. b indicates NC versus MDD. c indicates SD versus MDD. Tracking accuracy and tracking precision reflect eye-tracking data quality. NC, normal control. SD, subthreshold depression. MDD, major depressive disorder. BMI, body mass index. CES-D, Center for Epidemiologic Studies Depression Scale. HAMD-17, 17-item Hamilton Depression Rating Scale. GAD-7, 7-item Generalized Anxiety Disorder scale. HAMA-14, 14-item Hamilton Anxiety Rating Scale.

### 3.2 Multimodal fusion models

#### 3.2.1 Training dynamics and validation performance

Across both classification routes, Baseline-3 and Baseline-3+ showed rapid improvement in validation F1-macro across outer repeats during the early training phase, followed by a stable plateau, as shown in **Supplementary Figure 1**. Most gains occurred within approximately 5 to 10 epochs, indicating efficient convergence. Within Baseline-3, both Route A and Route B reached competitive performance quickly, although split-to-split variation remained visible. By contrast, Baseline-3+ showed faster early convergence, a higher plateau, and less fluctuation across outer repeats in both routes, suggesting that cross-modal interaction and training-stabilization strategies improved optimization stability.

#### 3.2.2 Overall outer-test performance

**Figure 2:**
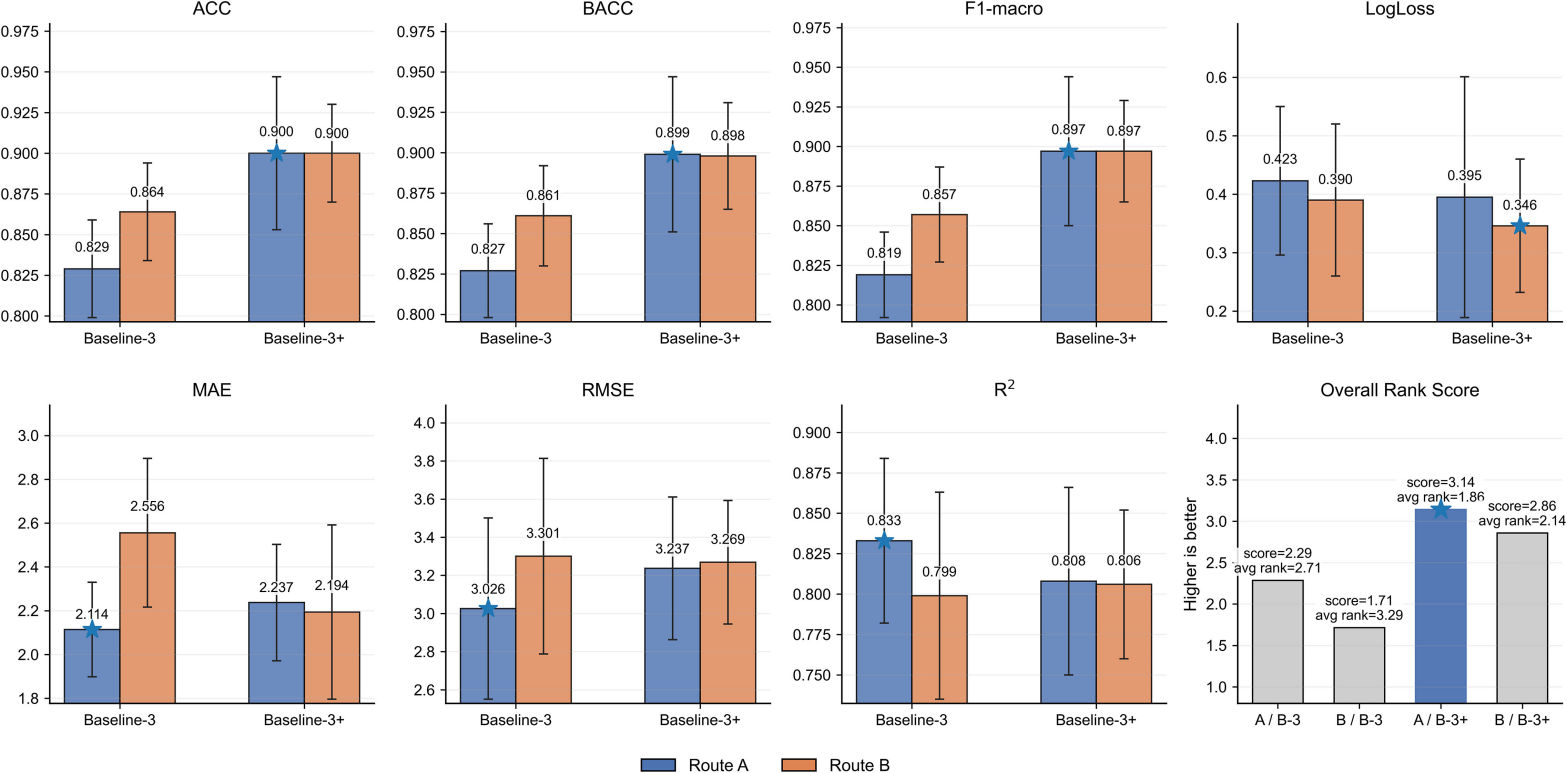
Outer-test performance of Baseline-3 and Baseline-3+ after temperature scaling. Bars show mean values with standard deviations across five outer-test repeats. Blue indicates Route A and orange indicates Route B. Classification performance is summarized by ACC, BACC, F1-macro, and log loss, and regression performance is summarized by MAE, RMSE, and R^2^ for HAMD-17 prediction. The composite-ranking panel provides an exploratory summary of the overall balance across performance metrics; it was included for visual comparison only and was not used for inferential comparison or model selection. ACC, accuracy; BACC, balanced accuracy; F1-macro, macro-averaged F1 score; HAMD-17, 17-item Hamilton Depression Rating Scale; MAE, mean absolute error; RMSE, root mean squared error; R^2^, coefficient of determination.

After temperature scaling, the outer-test results showed a clear performance structure across model families and classification routes, as shown in **Figure 2**.

For classification, Baseline-3+ generally improved threshold-dependent discrimination metrics, including ACC, BACC, and F1-macro, relative to Baseline-3. Within Baseline-3, Route B outperformed Route A across the main discrimination metrics. ACC improved from 0.829 ± 0.030 in Route A to 0.864 ± 0.030 in Route B. BACC improved from 0.827 ± 0.029 to 0.861 ± 0.031, and F1-macro improved from 0.819 ± 0.027 to 0.857 ± 0.030. Log loss was also lower in Route B, indicating better-calibrated class-probability fitting. Baseline-3+ further increased classification performance in both routes, with ACC, BACC, and F1-macro all approaching 0.90. The two routes were similar in point estimates, although Route B retained a lower log loss than Route A, suggesting an additional advantage in probabilistic output quality.

For regression, the pattern differed. Within Baseline-3, Route A provided more accurate continuous prediction of HAMD-17 severity than Route B, with lower MAE, lower RMSE, and higher R^2^. Within the original gated-fusion framework, the direct three-class organization appeared more favorable for continuous symptom estimation, whereas the hierarchical route preferentially benefited categorical discrimination. In Baseline-3+, this route-dependent difference became much smaller. MAE, RMSE, and R^2^ were closely similar between Route A and Route B, indicating that the introduction of cross-modal interaction and training-stabilization strategies attenuated the effect of classification-head structure on regression performance and partly compensated for the regression disadvantage previously seen in Route B. When classification, calibration, and regression metrics were considered jointly, Baseline-3+ showed the most balanced overall profile, with improved categorical performance and lower expected calibration error. The regression benefit was route-dependent and was most evident in reducing the regression disadvantage of the hierarchical route.

#### 3.2.3 Calibration and component-ablation analyses

Additional calibration analyses showed that Baseline-3+ achieved lower expected calibration error than Baseline-3 in both classification routes. Expected calibration error decreased from 0.149 to 0.069 in Route A and from 0.122 to 0.060 in Route B, indicating more reliable temperature-scaled class-probability estimates. Mean values with 95% confidence intervals and component-ablation results are provided in **Supplementary Table 3**, and the calibration summary is shown in **Supplementary Figure 2**. Full conventional benchmark results are provided in **Supplementary Table 4**. Exploratory paired outer-repeat sign-flip comparisons between Baseline-3+ and Baseline-3 are reported in **Supplementary Table 5**. These tests showed directionally favorable changes in the main classification metrics and expected calibration error, although improvements were not uniform across all metrics and most comparisons did not reach conventional statistical significance because only five matched outer repeats were available. Therefore, the paired tests were interpreted descriptively as repeat-consistency checks rather than confirmatory hypothesis tests. The component-ablation analysis further showed that the added Baseline-3+ components did not contribute uniformly to all outcomes. Removal of uncertainty-based dynamic task weighting produced the clearest deterioration in the regression component, especially under Route B. In contrast, removal of Transformer-based cross-modal interaction did not uniformly reduce categorical discrimination, suggesting that the quality-aware gated fusion module already captured substantial diagnostic information in the present dataset. The PCGrad sensitivity arm preserved a relatively favorable regression profile but did not improve categorical discrimination. Performance-oriented ablation effects relative to the full Baseline-3+ model are summarized in **Supplementary Figure 3**.

As prespecified regularized conventional benchmarks, Elastic-net logistic regression and Ridge regression provided tabular reference comparisons using single-modality and early-fusion feature sets. Early-fusion Elastic-net logistic regression achieved an ACC of 0.882, F1-macro of 0.884, and AUC-OVR of 0.958 for depressive-spectrum classification. Early-fusion Ridge regression achieved an MAE of 2.277, RMSE of 2.993, and R^2^ of 0.843 for HAMD-17 estimation. Notably, the face-only Elastic-net classifier also showed strong performance, consistent with the dominant contribution of facial behavior observed in the gating-weight and individual-attribution analyses. These benchmark results indicate that the extracted multimodal feature space, especially the facial-behavior feature block, contained substantial diagnostic and severity-related information. However, the conventional benchmarks should be interpreted as reference analyses of the extracted feature space rather than as replacements for the proposed quality-aware multitask framework, because they do not explicitly model modality quality, joint classification-regression learning, calibrated uncertainty profiling, or individual-level multimodal interpretation.

#### 3.2.4 Classification error patterns and class-level performance

**Figure 3:**
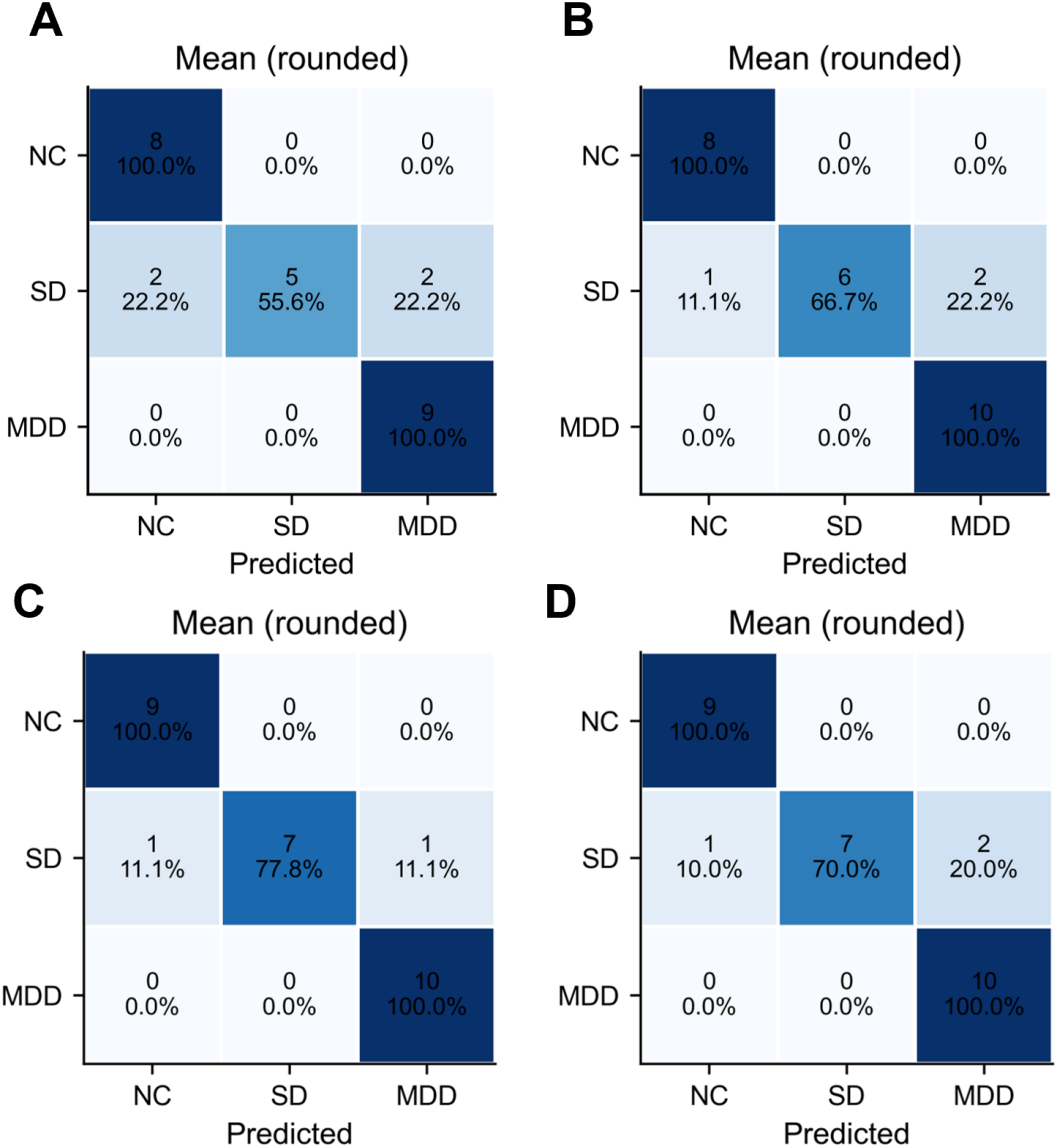
Mean row-normalized confusion matrices across outer-test repeats. Panels **(A, B)** show Baseline-3 under Route A and Route B, respectively, and Panels **(C, D)** show Baseline-3+ under Route A and Route B, respectively. Confusion matrices were summarized across five outer-test repeats. Each cell shows the mean rounded count together with the corresponding row-normalized percentage. Residual misclassification was concentrated mainly around the SD boundary. Counts are rounded for display; class recalls reported in the text and in **Supplementary Table 3** were calculated from repeat-wise row-normalized confusion matrices before rounding. MDD, major depressive disorder; NC, normal control; SD, subthreshold depression.

Confusion-matrix analysis showed that most classification errors occurred around the SD boundary, as shown in **Figure 3**. In Baseline-3, Route A classified the two extreme groups relatively well but showed weaker separation of SD. Mean class recall across outer-test repeats was approximately 93.3% for NC, 58.7% for SD, and 96.0% for MDD. Route B reduced this weakness: mean recall was approximately 91.1% for NC, 67.1% for SD, and 100% for MDD. This pattern was consistent with the higher ACC, BACC, and F1-macro of Route B, suggesting that the hierarchical design improved discrimination of the intermediate phenotype without compromising recognition of the extreme classes. In Baseline-3+, SD-related confusion was further reduced. Residual errors remained concentrated near the SD boundary, whereas recall for NC and MDD remained high under both routes. The remaining misclassifications were mainly limited to a small number of SD cases classified as NC or MDD, which is clinically plausible given the transitional and heterogeneous nature of SD.

#### 3.2.5 Continuous severity prediction

**Figure 4:**
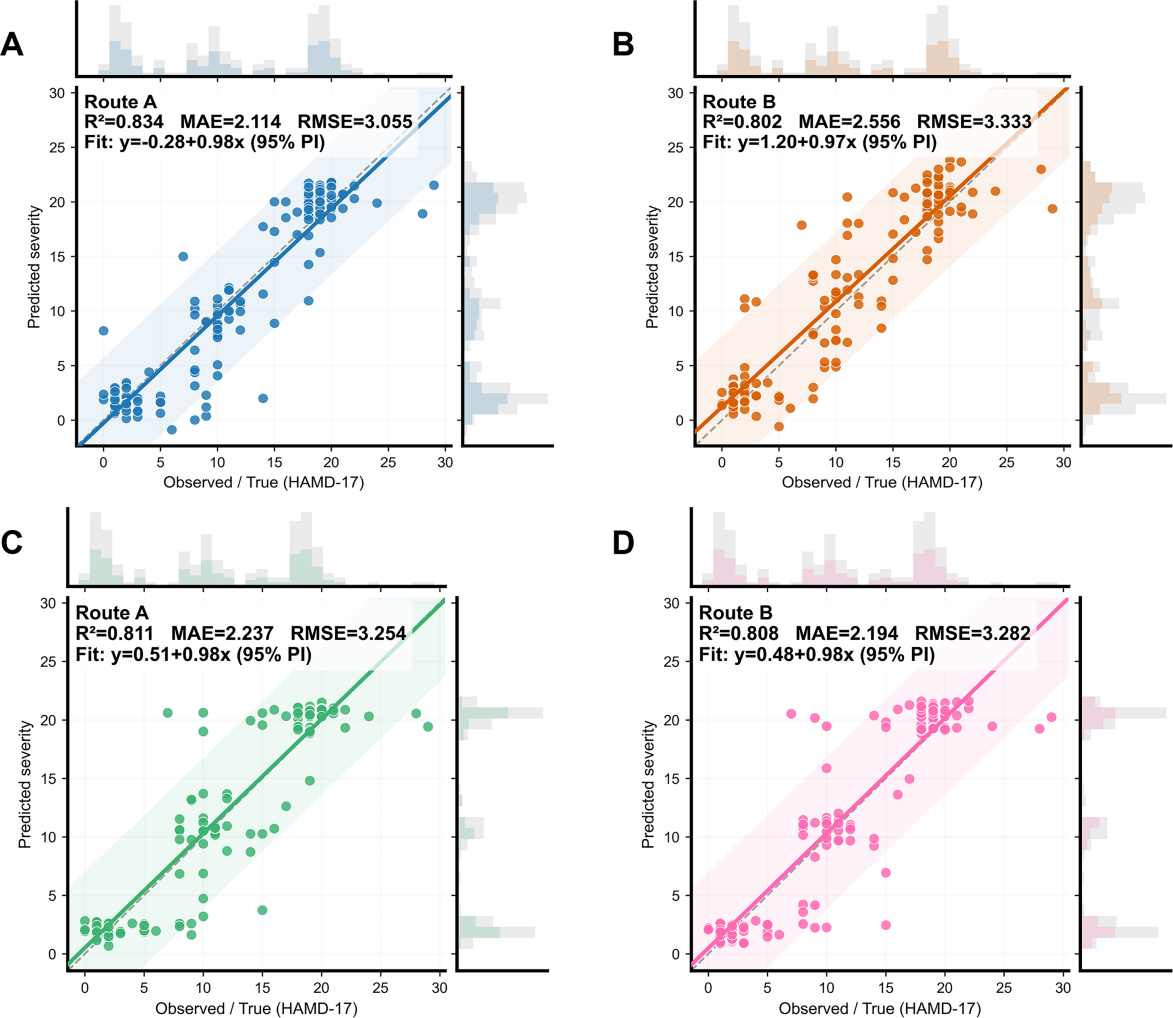
Regression fit and error distribution for HAMD-17 prediction. Panels **(A, B)** show Baseline-3 under Route A and Route B, respectively, and Panels **(C, D)** show Baseline-3+ under Route A and Route B, respectively. Scatterplots display observed and predicted HAMD-17 scores across the outer-test repeats. The dashed line indicates the line of identity, and the solid line indicates the fitted regression line. The shaded band represents the 95% prediction interval. Marginal histograms show the distributions of observed and predicted scores. R^2^, MAE, and RMSE are reported within each panel. HAMD-17, 17-item Hamilton Depression Rating Scale; MAE, mean absolute error; PI, prediction interval; R^2^, coefficient of determination; RMSE, root mean squared error.

Regression scatterplots showed generally good agreement between predicted and observed HAMD-17 scores across models and routes, as shown in **Figure 4**. Within Baseline-3, Route A provided the best overall fit. The fitted relationship was closely aligned with the identity line, with a slope near 1 and an intercept close to 0, indicating limited systematic bias and favorable calibration. Route B also preserved an approximately linear relationship but showed larger overall error and a positive intercept, suggesting mild overestimation in some score ranges. Within Baseline-3+, the regression difference between the two routes narrowed substantially. Both routes showed slopes close to 1 and only modest positive intercepts, indicating broadly comparable calibration. In both models, the 95% prediction interval widened at higher HAMD-17 scores, suggesting greater uncertainty in the high-severity range, consistent with greater clinical heterogeneity and lower sample density among individuals with more severe symptoms.

### 3.3 Model interpretability

#### 3.3.1 Quality-aware gating and cross-modal interaction

The gating-weight analysis showed that the multimodal fusion network learned stable modality-contribution profiles and adaptively reweighted modalities according to data completeness and signal quality, as shown in **Supplementary Figure 4** and **S5**. In Baseline-3, Route A relied more strongly on the facial modality, followed by eye tracking, with language receiving the smallest weight overall. Route B showed a more balanced allocation between facial and eye-tracking inputs, while language remained relatively weakly weighted. After stratification by diagnostic group, the general weighting pattern was preserved across NC, SD, and MDD, indicating that the learned modality preferences reflected broadly shared information value rather than a single class. After stratification by overall missingness tertiles, the high-missingness subgroup showed an expected shift toward more stable and more available modalities. In Baseline-3+, a similar overall gate-weight structure was observed, with facial signals remaining dominant and eye tracking ranking second. However, Transformer attention from the classification token to modality tokens showed relatively stronger allocation to language than was suggested by gate weights alone, indicating that language contributed to shared representation building through cross-modal interaction pathways.

#### 3.3.2 Confidence and selective prediction

Selective-prediction analysis showed that model confidence was clinically informative, as shown in **Supplementary Figure 6**. When only the highest-confidence cases were retained, classification accuracy remained high. As coverage increased and progressively lower-confidence cases were included, accuracy declined gradually. Although some fluctuation was observed at very low coverage because of the small retained sample size, the overall confidence-stratification pattern remained clear. Compared with Baseline-3, Baseline-3+ produced smoother coverage-accuracy curves across a wider coverage range, suggesting more reliable probability ranking.

#### 3.3.3 Drivers of classification errors

To identify factors associated with classification failure, an interpretable surrogate classifier was trained using prediction entropy, calibrated confidence, gate weights, modality missingness, availability indicators, and, for Baseline-3+, attention-derived features. In Baseline-3, these variables showed good ability to separate correct from incorrect predictions, with area under the receiver operating characteristic curve (AUC) values of 0.88 for Route A and 0.83 for Route B, as shown in **Supplementary Figure 7** and **S8**. These variables captured a coherent profile of error-prone cases. Across models, SHAP analyses consistently identified prediction entropy and calibrated confidence as the dominant error-related variables. Higher entropy was associated with increased error risk, whereas higher calibrated confidence was associated with reduced error risk. Beyond these uncertainty measures, language gate weight, eye-tracking missingness, and gate weights from the eye-tracking and facial modalities also contributed to classification-error risk, indicating that prediction instability increased when key modalities were degraded or incomplete. In Baseline-3+, surrogate discrimination was lower, with AUC values of 0.65 for Route A and 0.56 for Route B. After performance improvement, the remaining errors appeared fewer, more dispersed, or less well captured by simple uncertainty and quality descriptors. Nevertheless, SHAP ranking still highlighted uncertainty and calibrated confidence as the leading determinants, while attention-related variables, especially those involving language, also entered the important-feature set, suggesting a contribution of cross-modal interaction patterns to residual classification errors.

#### 3.3.4 Drivers of regression absolute error

A surrogate regression model was further used to explain absolute error in HAMD-17 prediction using the same family of uncertainty, confidence, gating, attention, and missingness variables, as shown in **Supplementary Figure 9** and **S10**. The overall explanatory power of these surrogate models was limited, indicating that variation in regression error was not fully captured by these quality and fusion descriptors alone. Even so, SHAP ranking showed a consistent pattern in which greater predictive uncertainty, lower calibrated confidence, and degradation of key modality quality were associated with larger absolute prediction errors.

### 3.4 Individual-level interpretability and case-based presentation

This section focuses on one representative Baseline-3+ Route B outer repeat selected for illustrative case-level interpretation. Although Baseline-3+ Route A showed a favorable overall metric profile, Route B was selected for detailed case-level interpretation because its hierarchical structure provided more clinically informative discrimination around the SD boundary, which represented the principal region of residual classification ambiguity. For illustrative case-level analysis, one representative outer repeat from Route B was examined in detail; this analysis was intended to demonstrate the interpretability workflow rather than to provide a population-level summary.

#### 3.4.1 Distribution of individual IG attributions

Across the held-out outer-test set of the representative Route B repeat, facial features dominated the attribution profile in 27 of 28 individuals, indicating that individual-level diagnostic decisions were driven primarily by facial dynamics and related behavioral signatures. Eye tracking often provided a secondary evidentiary channel, whereas language generally contributed smaller and more auxiliary signals. Among the most frequently selected high-attribution features, facial evidence was concentrated in action-unit-related intensity, duration, and transition measures. Recurrently important eye-tracking features included saccadic velocity and amplitude, fixation behavior, and area-of-interest (AOI) stability. Language features entered the top-ranking sets less frequently and with smaller absolute contributions, most often through length-related, distributional, or filler-density descriptors.

#### 3.4.2 Counterfactual analysis

Counterfactual analysis was performed with NC as the target class to evaluate local decision stability and reversibility under minimal perturbation. Overall, the target class was reached in 15 of 28 cases, corresponding to an achievement rate of 53.6%. However, success varied markedly by initial prediction. All cases initially predicted as NC already satisfied the target condition and required only minimal perturbation. Among cases initially predicted as SD, the achievement rate was 50.0%. Among cases initially predicted as MDD, no case could be shifted to NC within the imposed perturbation constraint. In cases where the target remained unattained, the target-class probability remained very low, particularly for MDD and a subset of SD cases, indicating greater local stability of the decision boundary around more severe phenotypes. Across cases, the largest counterfactual perturbation was usually observed in the facial modality, followed by eye tracking, with language showing the smallest magnitude of change. This pattern closely matched the IG results.

#### 3.4.3 Structured clinical summaries and representative cases

**Figure 5:**
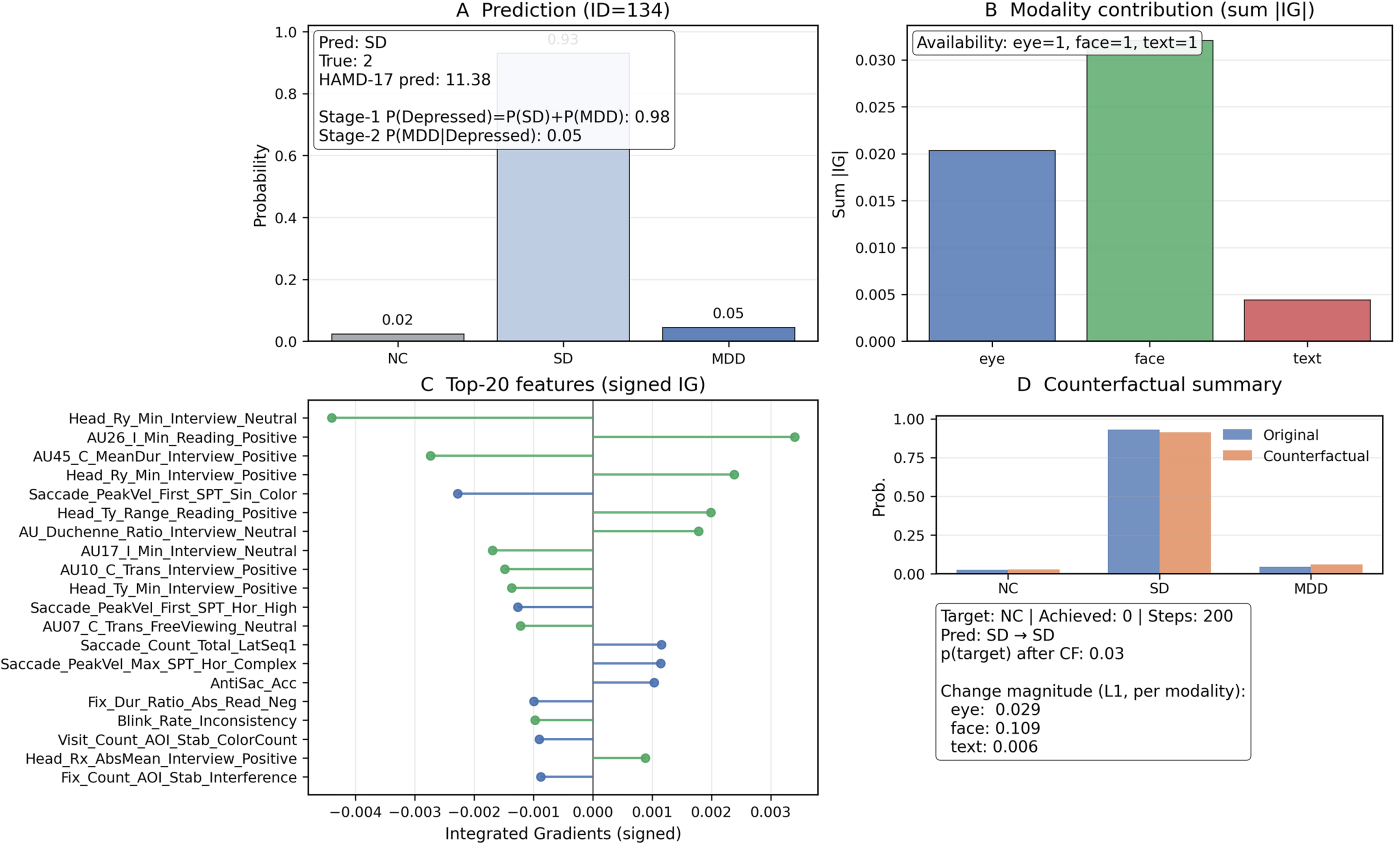
Individual-level interpretability for a representative case of subthreshold depression. This image presents the case-level interpretation generated by Baseline-3+ Route B for a representative participant with subthreshold depression. The panels summarize class probabilities, predicted HAMD-17 score, modality-level attribution magnitude, the leading Integrated Gradients features supporting the predicted class, and counterfactual analysis toward the NC target. The overall pattern is consistent with an intermediate position within the depressive spectrum, characterized by a high spectrum-level probability and a relatively low conditional probability of MDD. HAMD-17, 17-item Hamilton Depression Rating Scale; IG, Integrated Gradients; MDD, major depressive disorder; NC, normal control; SD, subthreshold depression.

For case-based presentation, class probabilities, HAMD-17 prediction, IG evidence, and counterfactual reversibility were integrated into structured case summaries. Within this 28-participant representative outer-test set, 11 cases showed low-risk profiles leaning toward NC, 8 showed intermediate-to-high-risk depressive-spectrum features, and 9 leaned toward MDD. Three representative cases illustrated low-risk, boundary, and high-risk profiles. The NC case showed high posterior probability for NC, a very low predicted HAMD-17 score, and near-zero counterfactual perturbation, indicating a stable low-risk classification. The SD case showed high spectrum-level probability but low conditional probability of MDD within the depressive spectrum, consistent with an intermediate position; the counterfactual target was not achieved within the perturbation limit. The MDD case showed high probability at both stages of the hierarchical route, a high predicted HAMD-17 score, and failure of counterfactual conversion to NC, indicating strong local stability of the high-risk classification. Representative visualizations are shown in **Figure 5** and **Supplementary Figure 11** and **S12**.

## 4 Discussion

In this single-cohort study, a quality-aware multimodal framework integrating eye tracking, facial behavior, and transcript-derived language showed favorable discrimination across NC, SD, and MDD while also supporting HAMD-17 severity estimation. The main contribution of this work is not limited to point-estimate performance. Instead, the framework integrates eye tracking, facial behavior, and transcript-derived language within a controlled task battery, explicitly models modality availability and missingness, combines quality-aware gated fusion with multitask learning, and evaluates calibrated prediction, component ablation, selective prediction, and case-level interpretability. Baseline-3+ showed the most favorable classification and calibration profile, whereas the hierarchical route most clearly improved discrimination of the intermediate phenotype, particularly SD. For HAMD-17 severity estimation, the benefit of Baseline-3+ was route-dependent and was most evident in reducing the regression disadvantage of the hierarchical route. Facial features contributed the largest share of explanatory signal, with complementary input from eye tracking and smaller but meaningful contributions from text through both direct and cross-modal pathways.

The concentration of misclassification around SD is clinically informative. This pattern is consistent with epidemiologic and clinical evidence that SD occupies an intermediate and heterogeneous position between health and syndromal depression (9–12). In our data, NC and MDD were classified more reliably than SD across model settings, and most residual errors involved SD being assigned to NC or MDD. The main challenge therefore appeared to lie not in global model instability but in ambiguity concentrated at the depressive-spectrum boundary. The improvement in SD recall under the hierarchical route suggests that a staged decision process is better aligned with the latent structure of depressive-spectrum phenotypes than a flat three-class decision. A clinically useful model may first need to distinguish depressive-spectrum status from nondepressed status and then refine subgroup discrimination within the spectrum. This structure may be particularly appropriate when the target phenotype is defined by partial symptom expression rather than by a sharply bounded syndrome.

The divergence between Route A and Route B in Baseline-3 further supports this interpretation. Within the original gated-fusion architecture, Route B improved classification, whereas Route A performed better for HAMD-17 regression. This suggests that shared representations optimized for boundary-sensitive categorical discrimination are not identical to those that best preserve continuous symptom gradients. After introducing cross-modal interaction and uncertainty-based dynamic task weighting in Baseline-3+, this route-dependent divergence became much smaller. These results suggest that the extended architecture improved compatibility between the classification and regression objectives, particularly by reducing the route-dependent regression disadvantage observed under the hierarchical setting.

The dominance of facial information is consistent with current evidence linking depression to reduced expressivity, altered facial affect, impaired emotional flexibility, and abnormalities in emotion recognition (20–25). In the present study, facial features were dominant in both gate-weight summaries and individual-level attributions. The convergence across interpretability levels suggests that facial behavior contributed distinct discriminative information rather than simply covarying with other modalities. One likely explanation lies in the structure of the task battery. Semi-structured interview and emotion-related description tasks place demands on spontaneous expression, interpersonal signaling, and behavioral regulation, domains in which depressive psychopathology is often clinically visible but difficult to quantify with conventional scales. These findings support the view that automated facial analysis can operationalize behavior that is familiar to clinicians but insufficiently standardized in routine assessment.

Eye tracking contributed differently. It was generally secondary to facial behavior in the fusion analyses, yet its contribution remained stable and became relatively more relevant in the hierarchical route. This pattern is compatible with literature linking depression to altered attentional allocation, affective bias, and oculomotor control across fixation, saccade, and pursuit paradigms (13–19). In our framework, eye-tracking signals may have contributed less to coarse separation of the extreme groups and more to resolving intermediate states. This interpretation is plausible because attentional bias and inhibitory control may vary gradually across the depressive spectrum and become especially informative near decision boundaries. The recurrent presence of eye-tracking features in individual attribution profiles further supports the view that they provided clinically meaningful, nonredundant information even when they were not the dominant modality.

The contribution of language differed from that of the other modalities. In the gate-weight analyses, language was consistently assigned lower weight than facial behavior or eye tracking. However, in Baseline-3+, attention analyses suggested a greater role for language in cross-modal interaction than gate weights alone would imply. This indicates that language functioned less as a primary standalone signal and more as a contextual or relational signal informing shared representation learning. Such a pattern is consistent with the present design. We modeled transcript-derived features rather than acoustic speech features, and the extracted variables were mainly lexical, structural, and task-conditioned. Under these conditions, language may provide only moderate standalone discrimination but still contribute meaningful disambiguation when integrated with behavioral channels. This interpretation is also consistent with the error analyses, in which language-related features remained relevant despite lower direct gate weight.

The observed advantages of Baseline-3+ likely reflected a combination of cross-modal interaction modeling, task-balance control, and improved calibration, rather than model capacity alone. The addition of explicit cross-modal interaction and uncertainty-based dynamic task weighting was associated with a smaller route-dependent difference in severity regression and improved the stability of outer-loop validation trajectories. The PCGrad sensitivity arm was used only to examine gradient-conflict mitigation under fixed task weighting and was not treated as part of the defining full Baseline-3+ setting (33–45). At the same time, our results indicate that such improvements are not uniform across tasks. In Baseline-3, Route A favored regression and Route B favored classification; in Baseline-3+, this tradeoff became less pronounced. This suggests that classification structure and shared representation learning interact in clinically relevant ways in multitask depressive-spectrum modeling.

The additional calibration and ablation analyses refine the interpretation of Baseline-3+. The extended architecture should not be viewed as uniformly superior across every metric and route. Its main advantage was reflected in improved classification, markedly lower expected calibration error, and a more balanced classification-regression profile compared with Baseline-3. Uncertainty-based dynamic task weighting appeared particularly relevant for preserving HAMD-17 regression performance under the hierarchical route, whereas Transformer-based cross-modal interaction did not provide uniform incremental benefit in this modest-sized cohort. This pattern is methodologically plausible because the quality-aware gated fusion module already captured strong modality-level signals, especially from facial behavior and eye tracking. Accordingly, the value of Baseline-3+ lies not only in point-estimate performance, but also in calibrated confidence, multitask balance, and richer modeling of multimodal interactions.

The prespecified conventional benchmarks further contextualize the proposed framework in relation to common early-fusion tabular modeling strategies used in depression-related machine learning. Their performance confirmed that the extracted multimodal feature space contained diagnostic and severity-related information. However, these models should be interpreted as reference analyses rather than competing replacements for the proposed framework. Regularized early-fusion models provide an interpretable estimate of the discriminative value of the feature space, but they do not explicitly model modality quality, do not jointly optimize depressive-spectrum classification and HAMD-17 regression, and do not provide calibrated uncertainty profiling or individual-level multimodal interpretation. In this sense, the contribution of Baseline-3+ is not limited to point-estimate performance. Its main value lies in linking incomplete multimodal evidence, multitask prediction, calibrated probability output, and case-level explanation within a single framework. This distinction is clinically relevant because the intended use case is not simple binary case detection, but stratification across NC, SD, and MDD with simultaneous severity estimation and interpretable evidence for boundary cases such as SD.

The uncertainty analyses are relevant to clinical translation because they identify cases in which model output should be interpreted with caution. Prediction entropy and calibrated confidence were the strongest determinants of classification error across models, and selective-prediction curves showed that confidence ranking enriched for more reliable predictions. In clinical settings, model utility depends not only on discrimination but also on the ability to identify cases in which output should be interpreted cautiously (3, 36–38, 42–44). The smoother coverage-accuracy curves in Baseline-3+ suggest that the extended model improved not only classification performance but also the internal ordering of predictive uncertainty. This is especially relevant for borderline cases, triage contexts, and workflows in which model output is intended to support rather than replace clinical judgment.

The surrogate error models help clarify what changed after performance improvement in Baseline-3+. In Baseline-3, uncertainty, confidence, and modality-quality descriptors separated correct from incorrect predictions relatively well. In Baseline-3+, this surrogate discrimination weakened. This is unlikely to mean that these variables became unimportant. Rather, once more systematic errors had been reduced, the remaining failures appeared fewer, more dispersed, and less easily summarized by a simple quality-and-uncertainty profile. This interpretation is consistent with the continued prominence of uncertainty and calibrated confidence in the SHAP rankings even after surrogate discrimination declined. It also suggests that future gains may depend less on general quality-aware fusion and more on targeted modeling of the residual boundary cases.

The individual-level explanation results were consistent with the overall model structure. In nearly all interpreted cases, facial features provided the dominant evidence, eye tracking contributed secondary evidence, and text served an auxiliary role. Counterfactual analysis further showed a gradient in local decision stability: low-risk NC cases were usually easy to preserve, MDD cases were much harder to move toward NC under constrained perturbation, and SD cases were intermediate. This pattern aligns with the group-level confusion structure and with the conceptual position of SD as a transition-prone but heterogeneous state. This pattern argues against a purely redundant fusion effect and supports clinically plausible organization of multimodal evidence.

Several limitations should be acknowledged. First, the sample was recruited from a single regional setting, and the cross-sectional design precludes direct inference about longitudinal transition from SD to MDD. The balanced group sizes facilitated model comparison but do not reflect clinical prevalence. External validation was not available, and future studies should evaluate prevalence-weighted performance in independent and naturalistic clinical cohorts. Acoustic speech features were not modeled as an independent modality, which likely narrowed the representational range of the language-related channel. Facial analysis depended on video quality and automated tracking, and language-feature extraction depended on automatic speech recognition, both of which may introduce systematic measurement error.

Second, HAMD-17 thresholds contributed to the operational definition of depressive-spectrum groups, and the HAMD-17 total score was also used as the regression target. The classification and severity-estimation tasks were therefore clinically related rather than statistically independent. The regression results should be interpreted as within-cohort estimation of the criterion symptom-severity scale, rather than as validation against an independent clinical endpoint. In addition, confidence intervals and paired tests were calculated across five outer repeats and should be viewed as descriptive resampling summaries rather than definitive inferential evidence. The Transformer extension increased model complexity and did not provide uniform incremental benefit across all outcomes, suggesting that simpler quality-aware gated-fusion models may remain preferable in settings where computational efficiency, interpretability, or deployment feasibility is prioritized. Finally, the interpretability analyses were intended to characterize model behavior rather than establish causal mechanisms of depressive psychopathology. These limitations indicate that the present framework should be viewed as a calibrated and interpretable research prototype for depressive-spectrum stratification rather than as a deployable diagnostic system.

## 5 Conclusions

Overall, a controlled multimodal task battery combined with explicit missingness modeling, calibrated prediction, and structured interpretation may support clinically meaningful stratification across the depressive spectrum. The most plausible role of this framework is to augment clinical assessment, particularly in intermediate or ambiguous cases such as SD, rather than to replace clinician judgment. Future work should prioritize external validation, longitudinal follow-up, integration of acoustic speech with transcript-derived language, and prospective evaluation in real clinical workflows.

## Data Availability

The de-identified feature-level data and custom code supporting the findings of this study will be made available by the corresponding author upon reasonable request. Raw facial-video, audio, transcript, and other potentially identifiable participant-level data are not publicly available because of participant privacy and ethical restrictions.
