# Supplementary material for: Multimodal behavioral phenotyping for depressive-spectrum classification and severity estimation using eye tracking, facial behavior, and transcript-derived language
Source: Front Psychiatry. 2026 Jun 16;17:1842005. doi: 10.3389/fpsyt.2026.1842005 (PMC13315236; doi:10.3389/fpsyt.2026.1842005)
Supplement: Supplementary file 1 [file Supplementaryfile1.docx]

**Supplementary Material**

**Appendix A. Eligibility criteria and group definitions**

Eligible participants were adults aged 18 to 80 years of either sex who had completed at least primary school education and had sufficient Mandarin reading ability to complete the experimental tasks.

Group definitions were prespecified before recruitment. The major depressive disorder (MDD) group met diagnostic criteria for depressive disorder according to the International Classification of Diseases, 11th Revision (ICD-11), and had a 17-item Hamilton Depression Rating Scale (HAMD-17) score greater than 17. The subthreshold depression (SD) group was operationally defined by a HAMD-17 score of 8 to 17 in participants who did not meet diagnostic criteria for major depressive disorder. The normal control (NC) group had no clinical diagnosis of depressive disorder and had a HAMD-17 score of 7 or lower. Participants were excluded if they had a severe medical illness likely to compromise study participation, schizophrenia spectrum disorders, bipolar disorder, depressive episodes with psychotic symptoms, intellectual disability, impaired consciousness, or substance abuse or dependence. Pregnancy and lactation were exclusion criteria. Recent acute infection, fever, or marked stress responses were also exclusionary. Conditions likely to compromise the validity of eye-tracking assessment were exclusionary, including clinically significant visual or hearing impairment, high myopia, color blindness, and ophthalmic disorders affecting visual function.

**Appendix B. Experimental setting and task administration**

All assessments were performed in a controlled laboratory environment to ensure comparability across eye-tracking, facial video, and transcript-derived text acquisition. Data collection took place in a separate quiet room. Participants were instructed to maintain a natural seated posture and to minimize large head movements.

Eye-tracking data were recorded using a Tobii Pro Nano eye tracker at 60 Hz. The device was mounted below a 14.5-inch laptop display with a screen resolution of 1920 × 1080 pixels. The viewing distance was maintained at approximately 60 to 70 cm. Facial videos were recorded with an external high-definition camera at 1080p and 30 frames per second. Spoken responses were transcribed for downstream feature extraction and quality control. Acoustic speech features were not used as an independent modeling modality in this study.

Before formal data collection, a nine-point calibration procedure was completed. Calibration was repeated until predefined acquisition quality was achieved. Standardized instructions were delivered by trained staff before each task, and one or two practice trials were provided when needed. A 30-second rest interval was arranged between tasks to reduce fatigue and carryover effects.

B1. Sociodemographic questionnaire

Sociodemographic, lifestyle, and psychosocial information was collected using a standardized questionnaire. Variables included demographic characteristics, body mass index (BMI), educational attainment, occupation, satisfaction with income, marital status, living arrangement, health-related behaviors, physical activity, social participation, and perceived emotional support.

B2. Semi-structured interview

The interview task comprised nine prompts spanning positive, neutral, and negative emotional contexts. Positive prompts invited participants to describe their hobbies or interests, recall a meaningful positive memory and its context, and discuss a favorite song, film, or television series together with its personal meaning. Neutral prompts addressed recent physical condition, hometown, and plans for the next three years. Negative prompts addressed recent sadness or hopelessness, thoughts occurring during sleeplessness, and emotional responses to misunderstanding with a close friend. Facial video was recorded continuously throughout the interview, and verbal responses were transcribed into text.

B3. Emotional text reading

Participants read aloud positive, neutral, and negative emotional word materials, with recording synchronized to stimulus presentation. Emotional words were selected from a standardized Chinese affective lexicon. Representative positive words included beauty, gentleness, friendship, bonus, praise, and blessing. Neutral words included ordinary, still, rule, train, adjustment, and emission. Negative words included vicious, contemptible, murderer, traffic accident, abuse, and fraud.

B4. Emotional free viewing

Participants viewed positive, neutral, and negative emotional images and were asked to describe either the image content or their subjective feelings. During interstimulus intervals, a black fixation point on a white background was presented at the screen center to provide a baseline. Stimuli included emotional face images with happy, calm, and sad expressions, as well as positive, neutral, and negative emotional scenes selected from standardized stimulus sets developed by Beijing Normal University on the basis of normative valence and arousal ratings. Eye-tracking and facial video were recorded synchronously, and spoken responses were transcribed into text. No fixed upper limit was imposed on viewing duration to preserve naturalistic visual behavior.

B5. Fixation stability task

Participants were instructed to maintain visual attention and continuously fixate a target stimulus with both eyes open.

In the simple condition, participants fixated a central black dot on a white screen for 10 seconds until the target disappeared. Two target sizes were tested, and each was repeated twice.

In the distractor condition, the central black target remained visible for 10 seconds while static distractor dots of the same size appeared in the background. Participants were instructed to ignore the distractors and maintain fixation on the central target. This condition was repeated twice.

In the color-counting condition, the target changed color in a random manner, and participants silently counted the number of color changes. This condition was repeated twice.

B6. Lateral gaze orienting task

A central fixation point was first presented as the baseline. Peripheral targets then appeared sequentially, and participants were instructed to shift gaze rapidly to each target and maintain stable fixation. Two target sequences were administered. The first followed center, left, up, right, and down and was repeated twice. The second followed center, right, down, left, and up and was also repeated twice. Each target remained visible for 3 seconds.

B7. Smooth pursuit task

This section included four horizontal smooth pursuit conditions and four sinusoidal smooth pursuit conditions. In all conditions, participants were instructed to continuously follow a moving target with their eyes.

For horizontal smooth pursuit, the target moved back and forth along the horizontal axis. The low-speed condition used a frequency of 0.2 Hz, and the high-speed condition used 0.4 Hz. In the complex-background condition, the target moved at 0.2 Hz while static distractor dots appeared in the background. In the color-counting condition, the target moved at 0.2 Hz while changing color, and participants silently counted the number of color changes. Each horizontal pursuit condition lasted 10 seconds.

For sinusoidal smooth pursuit, the target moved horizontally along a sinusoidal path. The low-speed and high-speed conditions used frequencies of 0.2 Hz and 0.4 Hz, respectively. Complex-background and color-counting conditions were implemented in the same manner as in horizontal pursuit. Each sinusoidal pursuit condition lasted 15 seconds.

B8. Prosaccade task

A central fixation point first appeared at the center of the screen and disappeared after 2 seconds. One second later, a peripheral target appeared at a random upper, lower, left, or right location and remained visible for 2 seconds. Participants were instructed to make a rapid saccade toward the target. Seven trials were administered.

B9. Antisaccade task

The antisaccade task used the same temporal structure as the prosaccade task. After the central fixation point disappeared and a peripheral target appeared, participants were instructed to suppress the reflexive saccade toward the target and instead voluntarily look in the opposite direction. Seven trials were administered.

**Appendix C. Modality-specific preprocessing and feature engineering**

C1. Eye-tracking modality

Raw eye-tracking samples were processed using the velocity-threshold identification algorithm to classify events into fixations, saccades, and blinks. Samples falling outside the stimulus display region were treated as invalid and excluded. The event-classification threshold was set at 30°/s. Blinks were identified on the basis of transient tracking loss and missing pupil information. Trials with a tracking ratio below 50% were removed at the quality-control stage.

After quality control, 723 initial eye-tracking features were extracted and aggregated at the participant level according to task paradigm, area of interest (AOI), and, where relevant, emotional condition. The feature space comprised fixation measures, visit measures, gaze-transition metrics, saccadic-control indices, and composite higher-order measures related to inhibitory control, interference cost, attentional bias, pupil-related arousal, and global scan behavior.

C2. Facial modality

Facial videos were processed frame by frame using OpenFace 2.0 to extract facial landmarks, head pose, and facial action unit (AU) features. Frames with confidence below 0.7 or failed tracking were discarded. At the segment level, recordings with a valid-frame ratio below 50% were excluded.

For continuous variables such as AU intensity and head-pose parameters, summary statistics including mean, standard deviation, maximum, minimum, and range were calculated. For binary AU presence, frequency, activation count, mean duration, and total duration were derived. Composite indices were then constructed to characterize overall facial activity, positive and negative affective expression, Duchenne-like smiling behavior, frown-to-smile balance, emotional reactivity, and task-related emotion regulation.

C3. Text modality

The text modality was derived from automatic transcription of spoken responses. Audio recordings were segmented by task and emotional condition and transcribed offline using Whisper large-v3 deployed through an Open Visual Inference and Neural Network Optimization (OpenVINO)-based workflow in Audacity. Because some tasks did not impose a fixed speaking duration, length-related variation was retained rather than truncated.

Text preprocessing included normalization of characters and punctuation, sentence segmentation, identification of short segments, and conservative token filtering. Only punctuation-only and blank tokens were removed. Chinese word segmentation was performed using the precise mode of jieba. Features were computed at the segment level and then aggregated at the participant level within each task and emotional condition.

The final text feature set included completeness and quality indicators, text length and structural measures, expressive-style and fluency indices, clinically relevant linguistic markers such as negation, self-reference, and absolutist wording, character-composition measures, and affective lexical features derived from the Dalian University of Technology Chinese Emotion Ontology lexicon. Cross-condition contrast features were further constructed to quantify within-subject shifts in expression across emotional contexts.

**Appendix D. Data alignment, missingness handling, and model development**

Tabular features from the eye-tracking, facial, and text modalities were read from separate files and aligned by a unique participant identifier after duplicate checking and index harmonization. The resulting reproducible master table contained 186 participants, including 62 NC, 62 SD, and 62 MDD participants. It included 723 eye-tracking features, 2021 facial-behavior features, and 1029 transcript-derived language features. Cross-table consistency checks identified no conflicts in diagnosis labels or HAMD-17 scores. Complete three-modality data were available for 168 participants, whereas 18 participants had at least one unavailable modality. The master-table construction and modality-availability patterns are reported in Supplementary Tables S1 and S2.

The classification target was depressive-status group membership, defined as NC, SD, or MDD. The regression target was the HAMD-17 total score. When label fields were missing in modality-specific tables, available non-missing labels were retained after cross-source consistency checking. Unresolved label conflicts were excluded after manual review.

To model incomplete multimodal acquisition, modality-level meta-features were constructed, including availability indicators and modality-specific missing ratios. After fold-specific preprocessing, the entire feature block of an unavailable modality was set to zero so that the model could distinguish complete modality absence from partial within-modality missingness.

Model development followed a nested repeated-resampling design. In the outer loop, data were repeatedly divided into training and held-out test sets using stratified random splitting with an 85% to 15% ratio over five repeats. In the inner loop, five-fold stratified cross-validation was used within each outer-training partition for hyperparameter selection and early stopping.

Within each inner-loop training fold, preprocessing was applied independently to each modality. Features with more than 20% missingness were removed. Remaining features were median-imputed and winsorized at the 1st and 99th percentiles. Near-zero-variance features were removed using a coefficient of variation (CV) threshold of 0.01. Redundant features were pruned using the absolute Spearman correlation matrix with a threshold of 0.95. Retained features were standardized using z transformation based on training-fold statistics only.

After preprocessing, supervised feature selection was performed within each modality by repeated subsampling stability selection. A multinomial logistic regression model with elastic-net regularization and balanced class weights was fitted across 60 repeated subsamples, each using 80% of the training data without replacement. Features with a non-zero coefficient in at least 60% of subsamples were retained. Lower and upper bounds were applied to stabilize feature count. Mutual information was used as a fallback selection strategy when stability selection did not yield a valid feature set. Meta-features were retained throughout this step.

**Appendix E. Multimodal fusion models, evaluation, and interpretability**

The primary multimodal model, termed Baseline-3, used modality-specific multilayer perceptron (MLP) encoders to transform each modality into a fixed-dimensional embedding. A quality-aware gating module generated fusion weights on the basis of modality availability and missingness, allowing adaptive weighting under variable data quality and missing-modality patterns. A shared latent representation was subsequently passed to separate classification and regression heads.

Mathematical formulation of the multimodal fusion framework

Let $x_{m}$ denote the preprocessed feature vector for modality $m$, where $m\in\left\{ eye, face, text \right\}$. Each modality was encoded using a modality-specific multilayer perceptron:

$$h_{m}=f_{m}\left( x_{m} \right).$$

Modality-availability indicators and modality-specific missingness ratios were summarized as quality metadata $q_{m}$. The quality-aware gating module generated modality scores $g_{m}$ from $h_{m}$ and $q_{m}$, followed by softmax normalization:

$$\alpha_{m}=\frac{exp\left( g_{m} \right)}{\sum_{k} exp\left( g_{k} \right)}.$$

The Baseline-3 fused representation was obtained as a weighted combination of modality embeddings:

$$h=\sum_{m} \alpha_{m}h_{m}.$$

The fused representation was passed to a classification head and a regression head:

$$\hat{y}_{cls}=F_{cls}\left( h \right), \hat{y}_{reg}=F_{reg}\left( h \right).$$

For Route A, the classification head directly estimated the three-class probability distribution over NC, SD, and MDD. For Route B, the hierarchical route first estimated the probability of depressive-spectrum status and then estimated the conditional probability of MDD within the depressive spectrum. The final class probabilities were computed as:

$$P\left( NC \right)=1-P\left( Spectrum \right),$$

$$P\left( SD \right)=P\left( Spectrum \right)\left\{ 1-P\left( MDD | Spectrum \right) \right\},$$

$$P\left( MDD \right)=P\left( Spectrum \right)P\left( MDD | Spectrum \right).$$

In Baseline-3+, modality embeddings were treated as modality tokens and passed through a lightweight Transformer encoder:

$$\tilde{H}=Transformer\left( H \right),$$

where $H=\left[ h_{eye}, h_{face}, h_{text} \right]$ denotes the stacked modality-token representation. The resulting interaction-aware representation was then used for the classification and regression heads.

The multitask objective combined classification and regression losses. For fixed task weighting, the loss was:

$$\mathcal{L=}\mathcal{L}_{cls}+{\lambda\mathcal{L}}_{reg}.$$

In Baseline-3+, uncertainty-based dynamic task weighting introduced learnable task-uncertainty parameters $s_{c}$ and $s_{r}$:

$$\mathcal{L=}\exp\left( -s_{c} \right)\mathcal{L}_{cls}+s_{c}+\exp\left( -s_{r} \right)\mathcal{L}_{reg}+s_{r}.$$

Temperature scaling was used for post hoc probability calibration. Given logits z and temperature parameter T, calibrated probabilities were calculated as:

$$P\left( y=c|x \right)=\frac{exp(z_{c}/T)}{\sum_{j} exp(z_{j}/T)}$$

Expected calibration error was calculated by comparing mean confidence and empirical accuracy across confidence bins:

$$ECE=\sum_{b=1}^{B} \frac{|B_{b}|}{n}\left| acc\left( B_{b} \right)-conf\left( B_{b} \right) \right|.$$

PCGrad was evaluated separately as a sensitivity arm under fixed task weighting and was therefore not combined with uncertainty-based task weighting in the full Baseline-3+ setting.

Model training used modality dropout, AdamW optimization, and gradient clipping. Modality dropout was applied during model training only. At each training iteration, available modality embeddings could be randomly masked according to the modality-dropout hyperparameter selected within the inner cross-validation loop. Validation and outer-test evaluation used the observed modality-availability pattern without artificial dropout. Regression targets were standardized within the training data of each split and inverse-transformed for evaluation.

Classification performance was assessed using accuracy (ACC), balanced accuracy (BACC), one-vs-rest macro-averaged area under the receiver operating characteristic curve (AUC-OVR), macro-averaged F1 score (F1-macro), and log loss. Regression performance was assessed using mean absolute error (MAE), root mean squared error (RMSE), and coefficient of determination (R^2^). Metrics were calculated on the outer-loop test sets and summarized across repeated splits as mean ± standard deviation.

Interpretability analyses were performed at both the modality and individual levels. Gating-weight distributions were examined overall, by diagnostic group, and by missingness burden. Individual-level feature attribution was quantified using Integrated Gradients (IG). Counterfactual explanations were generated under modality-availability constraints to identify plausible input changes associated with altered predictions and to support case-level clinical interpretation.

**Appendix F. Additional calibration, component-ablation, and conventional benchmark analyses**

To provide additional evidence on calibration, resampling stability, model-component contribution, and representative conventional comparisons, we supplemented the outer-test evaluation with expected calibration error, 95% confidence intervals, paired outer-repeat comparisons, component-ablation analyses, and prespecified regularized conventional benchmarks. Expected calibration error was calculated from temperature-scaled class probabilities using 10 equal-width confidence bins. Full Baseline-3 and full Baseline-3+ were retained as reference settings. Additional ablation settings selectively removed uncertainty-based dynamic task weighting or Transformer-based cross-modal interaction, and a PCGrad sensitivity arm was evaluated under fixed task weighting. Conventional benchmark analyses used Elastic-net logistic regression for depressive-spectrum classification and Ridge regression for HAMD-17 severity estimation. These models were used as prespecified regularized tabular references, including single-modality and early-fusion feature sets, because they are interpretable, regularized, and suitable for high-dimensional small-sample data. They were not intended to reproduce deep audiovisual, speech-language, or attention-based multimodal architectures. Full-model and component-ablation results are summarized in Supplementary Table S3, prespecified conventional benchmark results are summarized in Supplementary Table S4, and exploratory paired outer-repeat sign-flip comparisons are reported in Supplementary Table S5. Calibration and ablation-effect summaries are shown in Supplementary Figures S2 and S3.

| 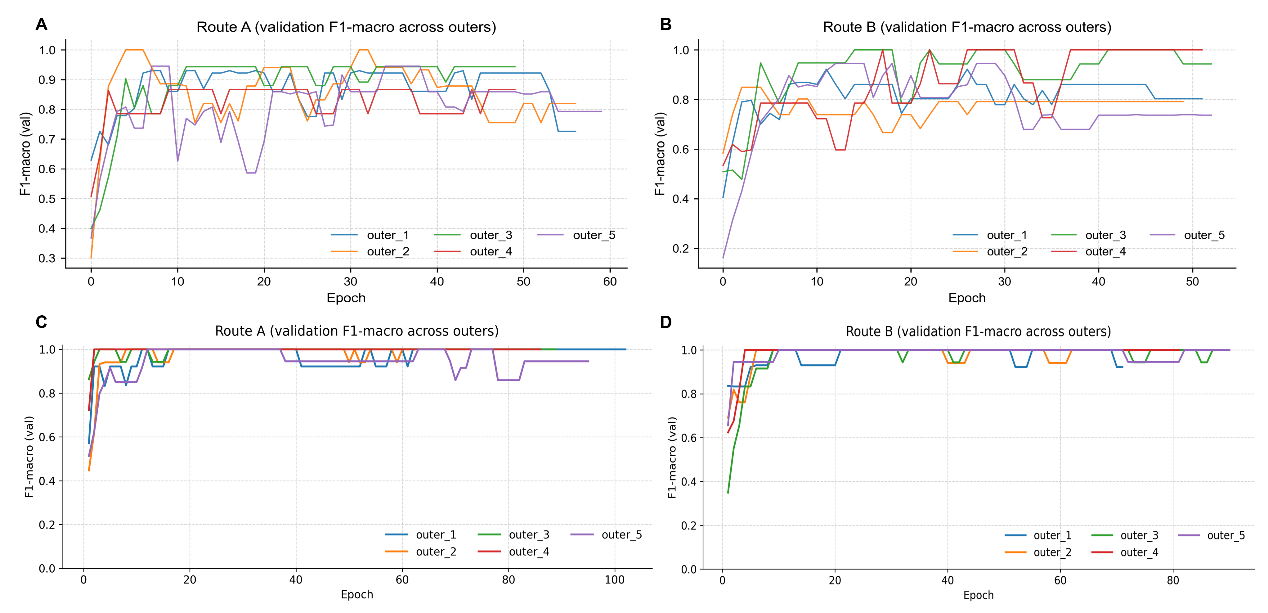 |
| --- |

**Supplementary Figure S1. Validation F1-macro trajectories across training epochs**

Legend. Panels A and B show Baseline-3 under Route A and Route B, respectively, and Panels C and D show Baseline-3+ under Route A and Route B, respectively. Curves show validation F1-macro across training epochs for five outer-test repeats. In all settings, performance improved rapidly during early training and then approached a stable plateau. Abbreviations: F1-macro, macro-averaged F1 score.

| 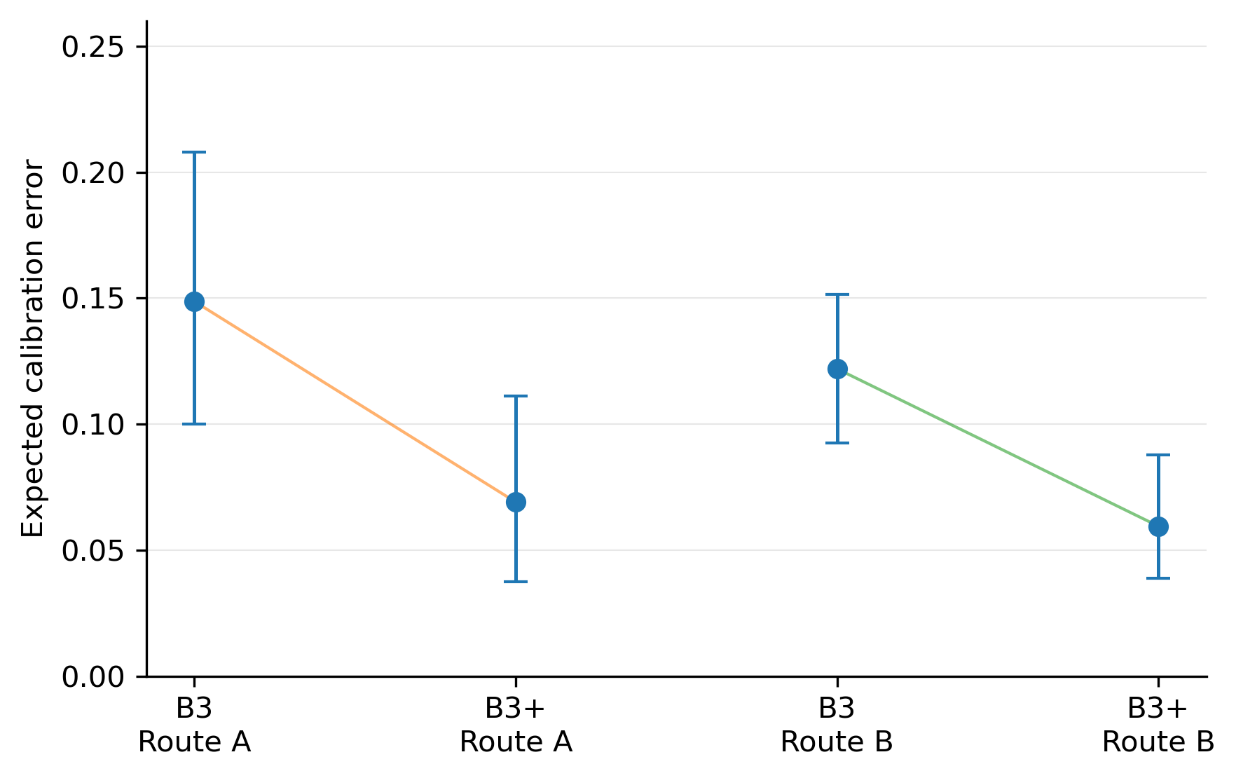 |
| --- |

**Supplementary Figure S2. Expected calibration error of Baseline-3 and Baseline-3+ across classification routes**

Legend. Points show mean expected calibration error across five outer-test repeats, and error bars indicate 95% confidence intervals. Expected calibration error was calculated from temperature-scaled class probabilities using 10 equal-width confidence bins. Lower values indicate better probability calibration. Abbreviations: ECE, expected calibration error.

| 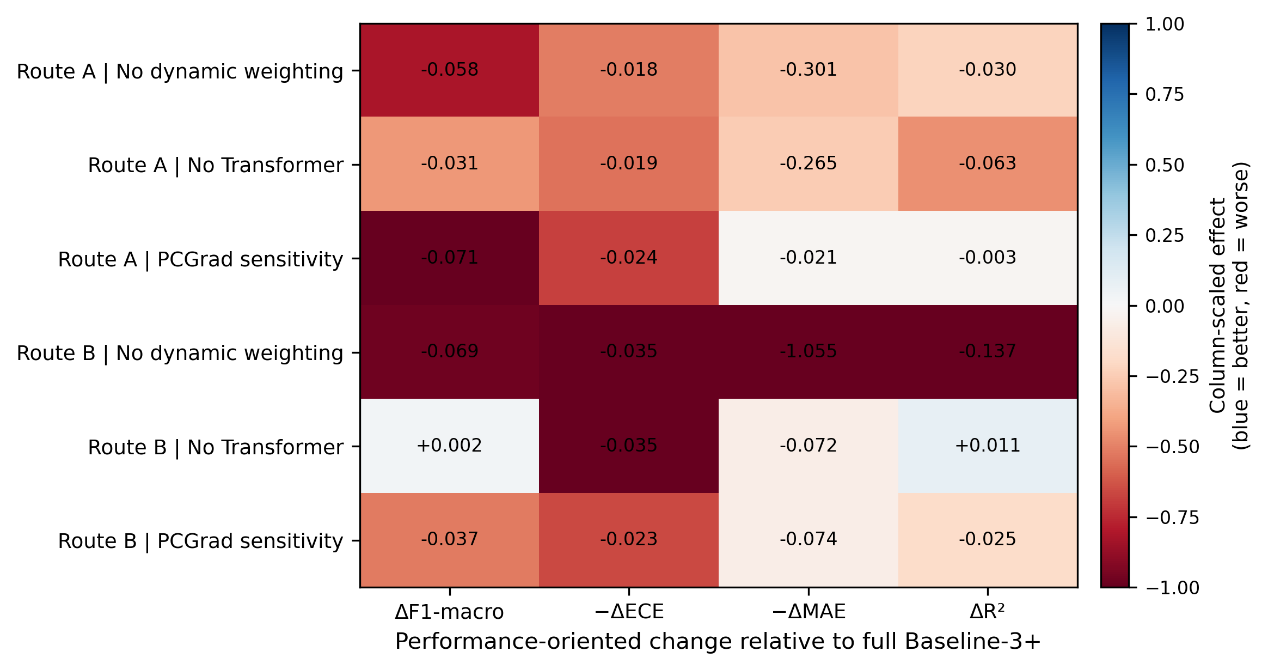 |
| --- |

**Supplementary Figure S3. Component-ablation effects relative to the full Baseline-3+ model**

Legend. Cell values indicate performance-oriented differences between each ablation or sensitivity setting and the full Baseline-3+ reference within the same route after metric-direction alignment. Positive values indicate better performance for the ablation or sensitivity setting after direction alignment. For ECE and MAE, differences were sign-adjusted so that positive values consistently indicate improvement. Thus, positive values correspond to higher F1-macro, lower ECE, lower MAE, or higher R^2^. PCGrad was evaluated as a sensitivity arm under fixed task weighting. Abbreviations: ECE, expected calibration error; F1-macro, macro-averaged F1 score; MAE, mean absolute error; PCGrad, Projected Conflicting Gradient; R^2^, coefficient of determination.

| 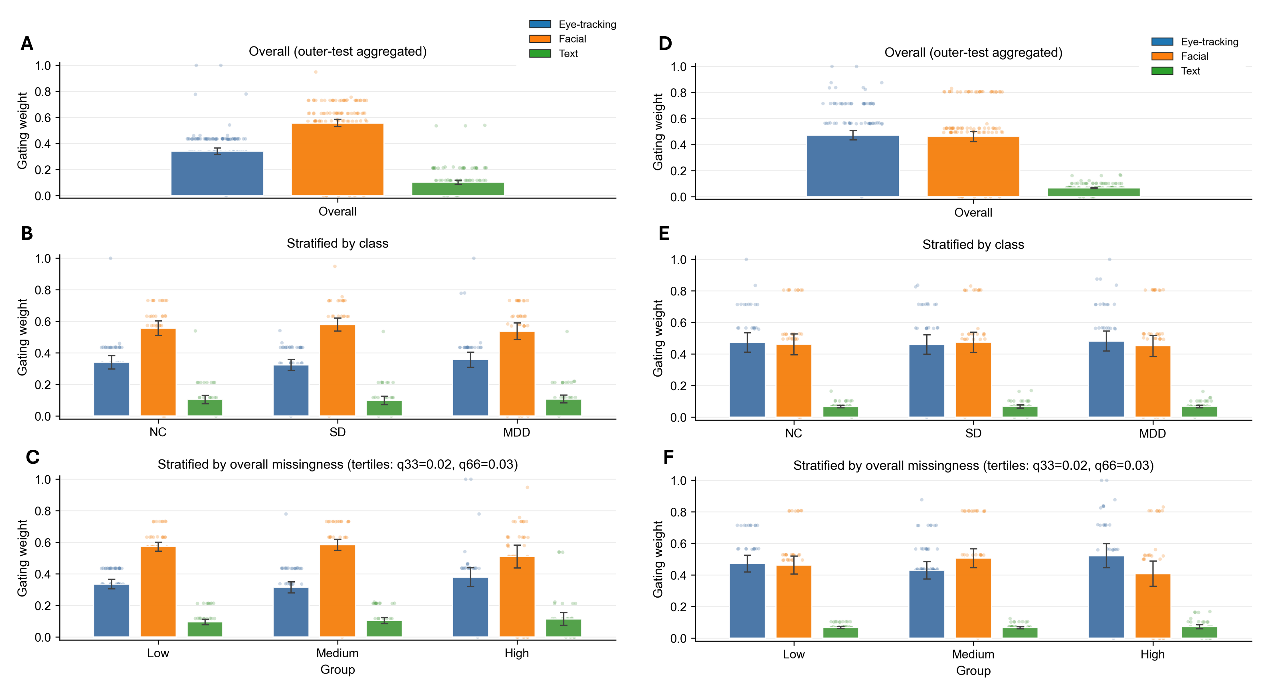 |
| --- |

**Supplementary Figure S4. Quality-aware gating patterns in Baseline-3**

Legend. Panels A to C correspond to Route A, and Panels D to F correspond to Route B. The figure shows overall gate-weight distributions and summaries stratified by diagnostic group and overall missingness tertile. Error bars indicate standard deviations across outer-test repeats where applicable. Abbreviations: MDD, major depressive disorder; NC, normal control; SD, subthreshold depression.

| 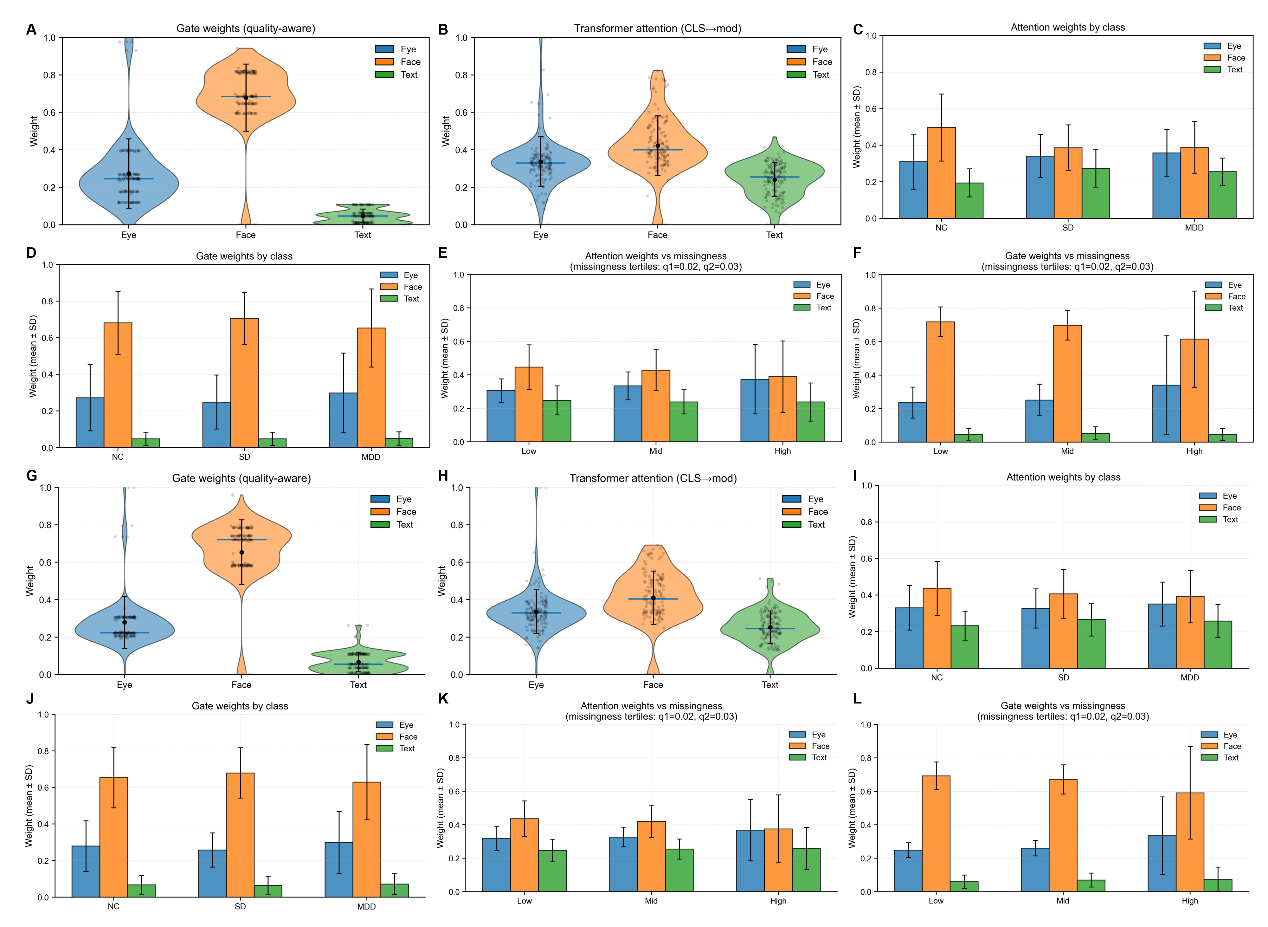 |
| --- |

**Supplementary Figure S5. Quality-aware gating and cross-modal interaction in Baseline-3+**

Legend. Panels A to F correspond to Route A, and Panels G to L correspond to Route B. The figure shows overall gate-weight distributions, attention distributions from the classification token to modality tokens, and summaries stratified by diagnostic group and overall missingness tertile. Abbreviations: MDD, major depressive disorder; NC, normal control; SD, subthreshold depression.

| 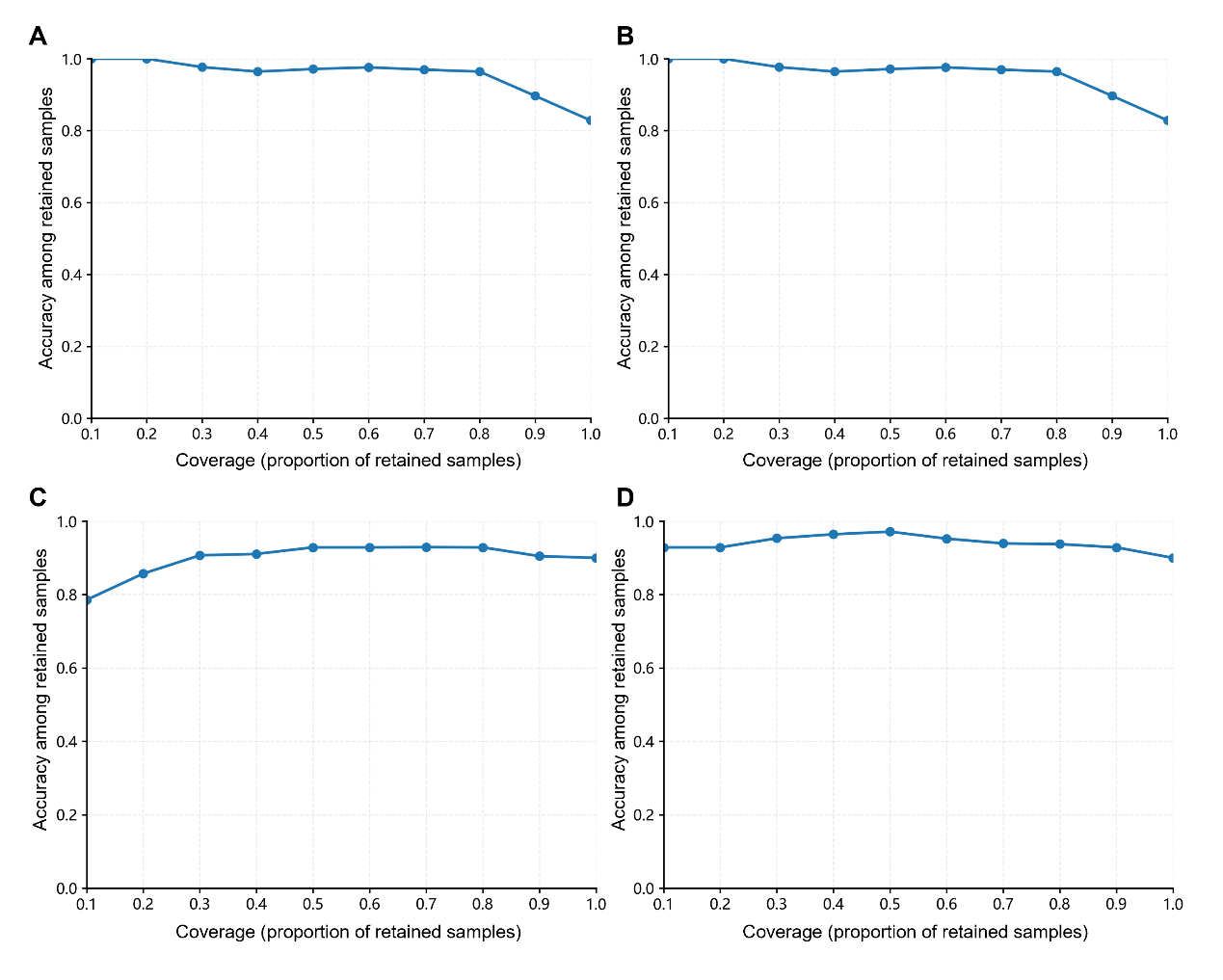 |
| --- |

**Supplementary Figure S6. Coverage-accuracy curves for selective prediction based on calibrated confidence**

Legend. Panels A and B show Baseline-3 under Route A and Route B, respectively, and Panels C and D show Baseline-3+ under Route A and Route B, respectively. Samples were ranked by maximum temperature-scaled class probability. ACC was calculated among retained samples at each coverage level. Higher accuracy at lower coverage indicates enrichment for more reliable predictions by calibrated confidence. Abbreviations: ACC, accuracy.

| 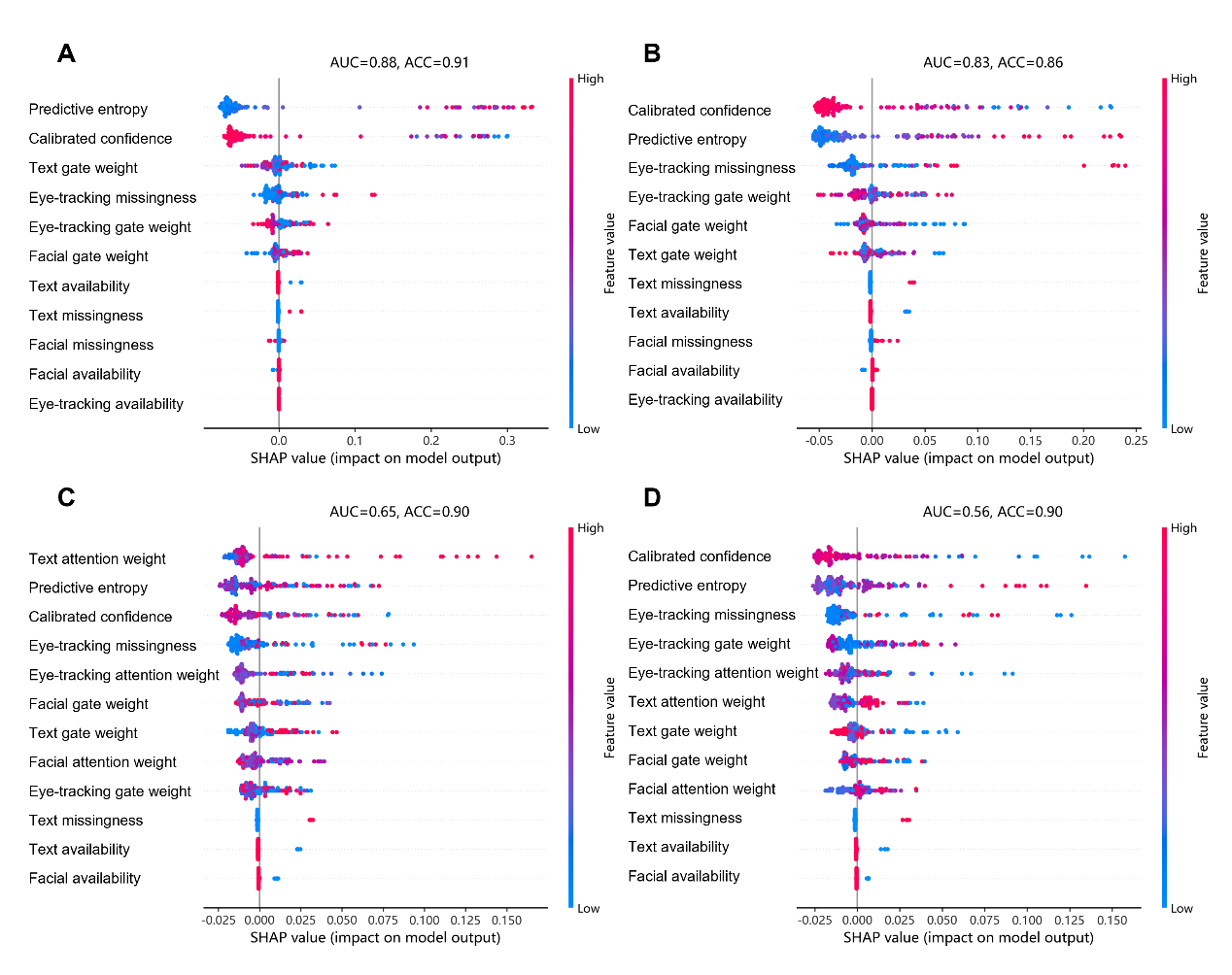 |
| --- |

**Supplementary Figure S7. SHAP beeswarm plots for surrogate models of classification error**

Legend. Panels A and B show Baseline-3 under Route A and Route B, respectively, and Panels C and D show Baseline-3+ under Route A and Route B, respectively. Surrogate classifiers were trained to distinguish incorrect from correct predictions using predictive entropy, calibrated confidence, gate weights, modality missingness, availability indicators, and attention-derived features for Baseline-3+. Point color indicates feature value from low to high. Positive SHAP values indicate increased error risk. AUC and ACC are shown within each panel. Abbreviations: ACC, accuracy; AUC, area under the receiver operating characteristic curve; SHAP, SHapley Additive exPlanations.


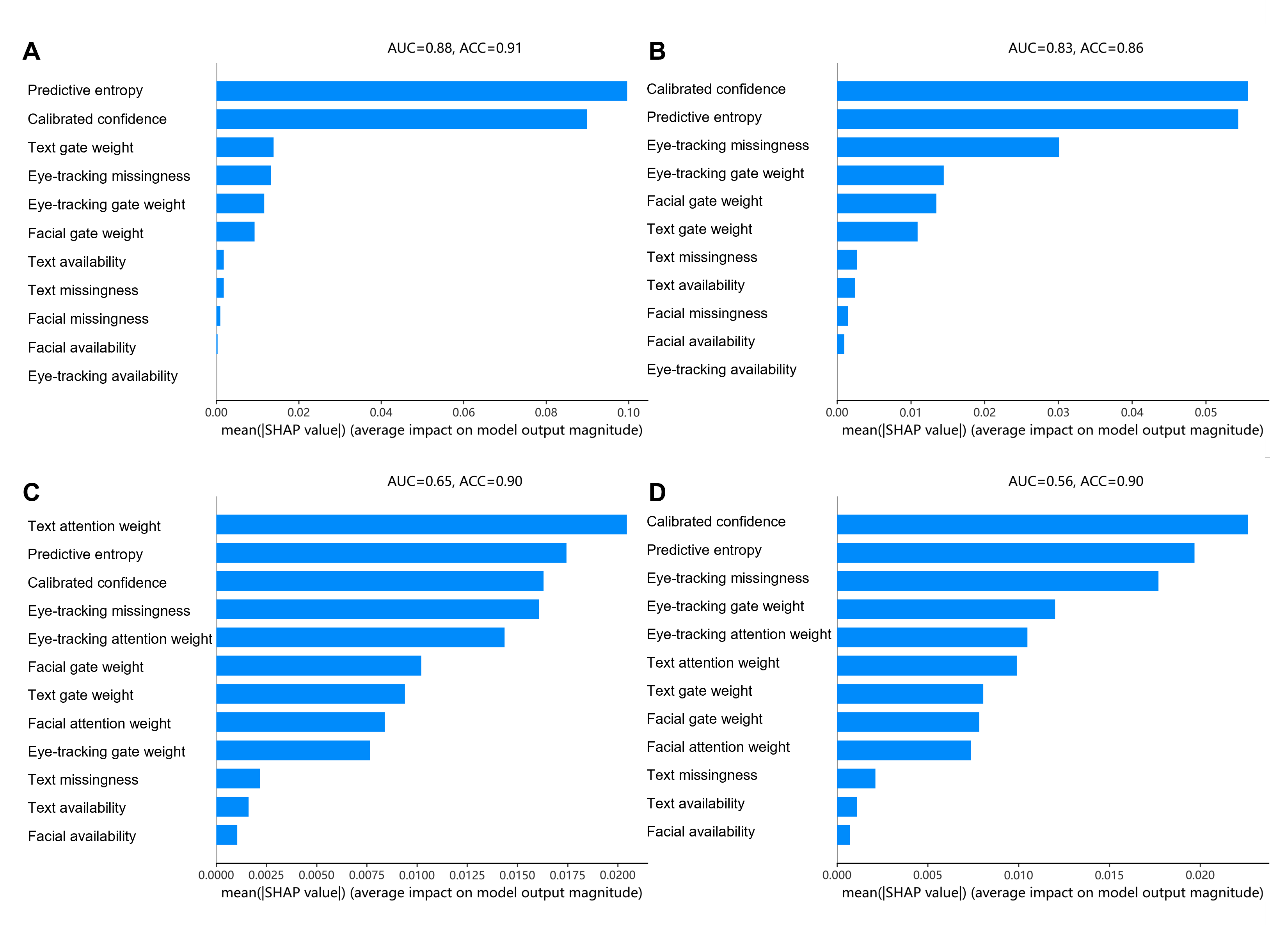


**Supplementary Figure S8. Global SHAP importance for surrogate models of classification error**

Legend. Panels A and B show Baseline-3 under Route A and Route B, respectively, and Panels C and D show Baseline-3+ under Route A and Route B, respectively. Bars represent mean absolute SHAP values and summarize the average contribution of each feature to the surrogate classification-error model. AUC and ACC are reported within each panel. Abbreviations: ACC, accuracy; AUC, area under the receiver operating characteristic curve; SHAP, SHapley Additive exPlanations.

| 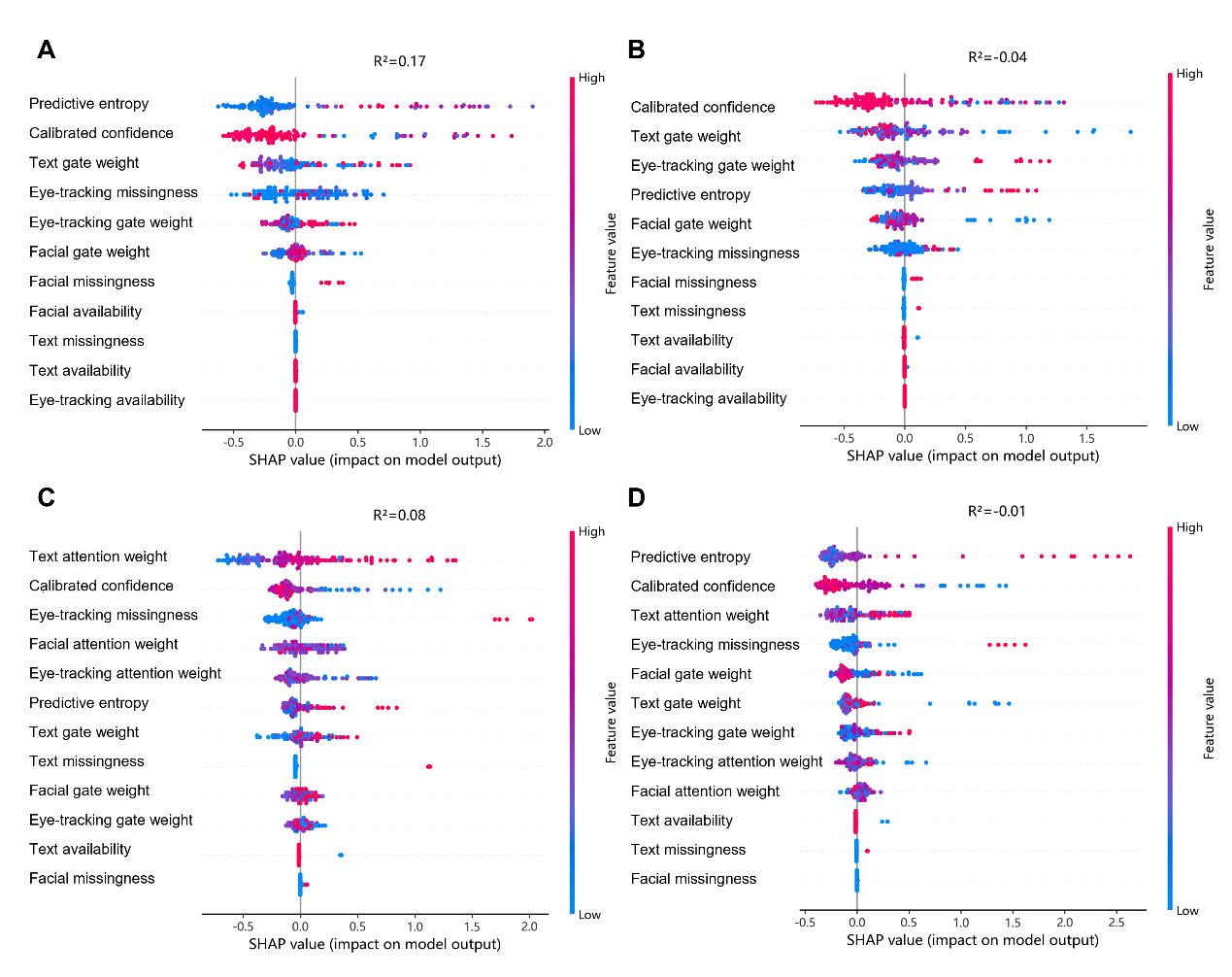 |
| --- |

**Supplementary Figure S9. SHAP beeswarm plots for surrogate models of regression absolute error**

Legend. Panels A and B show Baseline-3 under Route A and Route B, respectively, and Panels C and D show Baseline-3+ under Route A and Route B, respectively. Surrogate regressors were trained to predict absolute HAMD-17 error using predictive entropy, calibrated confidence, gate weights, modality missingness, availability indicators, and attention-derived features for Baseline-3+. Point color indicates feature value from low to high. Positive SHAP values indicate factors associated with larger absolute error. R^2^ is reported within each panel. Abbreviations: HAMD-17, 17-item Hamilton Depression Rating Scale; R^2^, coefficient of determination; SHAP, SHapley Additive exPlanations.

| 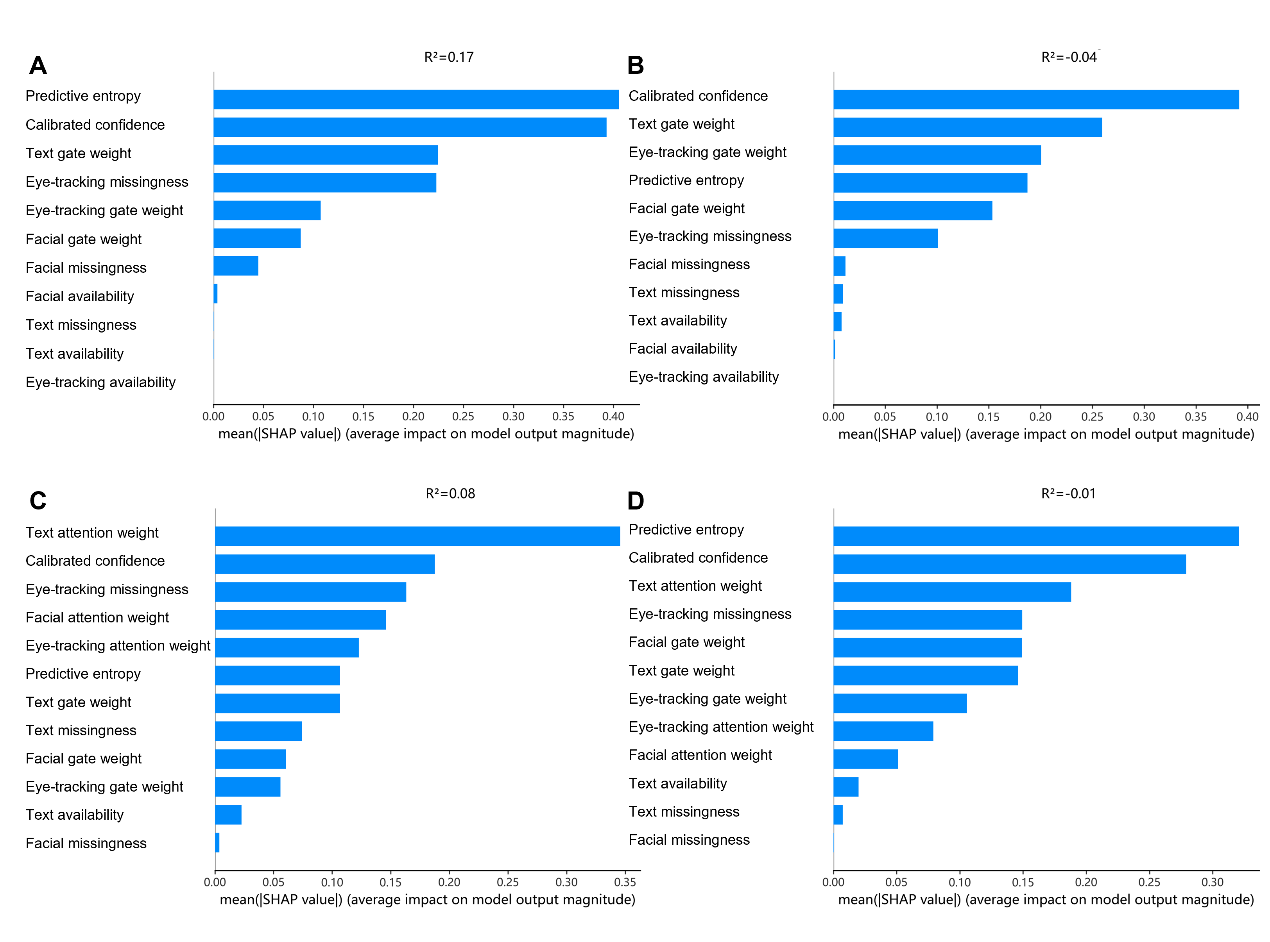 |
| --- |

**Supplementary Figure S10. Global SHAP importance for surrogate models of regression absolute error**

Legend. Panels A and B show Baseline-3 under Route A and Route B, respectively, and Panels C and D show Baseline-3+ under Route A and Route B, respectively. Bars represent mean absolute SHAP values and summarize the global importance of uncertainty, confidence, fusion, and data-quality variables in relation to absolute HAMD-17 prediction error. R^2^ is reported within each panel. Abbreviations: HAMD-17, 17-item Hamilton Depression Rating Scale; R^2^, coefficient of determination; SHAP, SHapley Additive exPlanations.

| 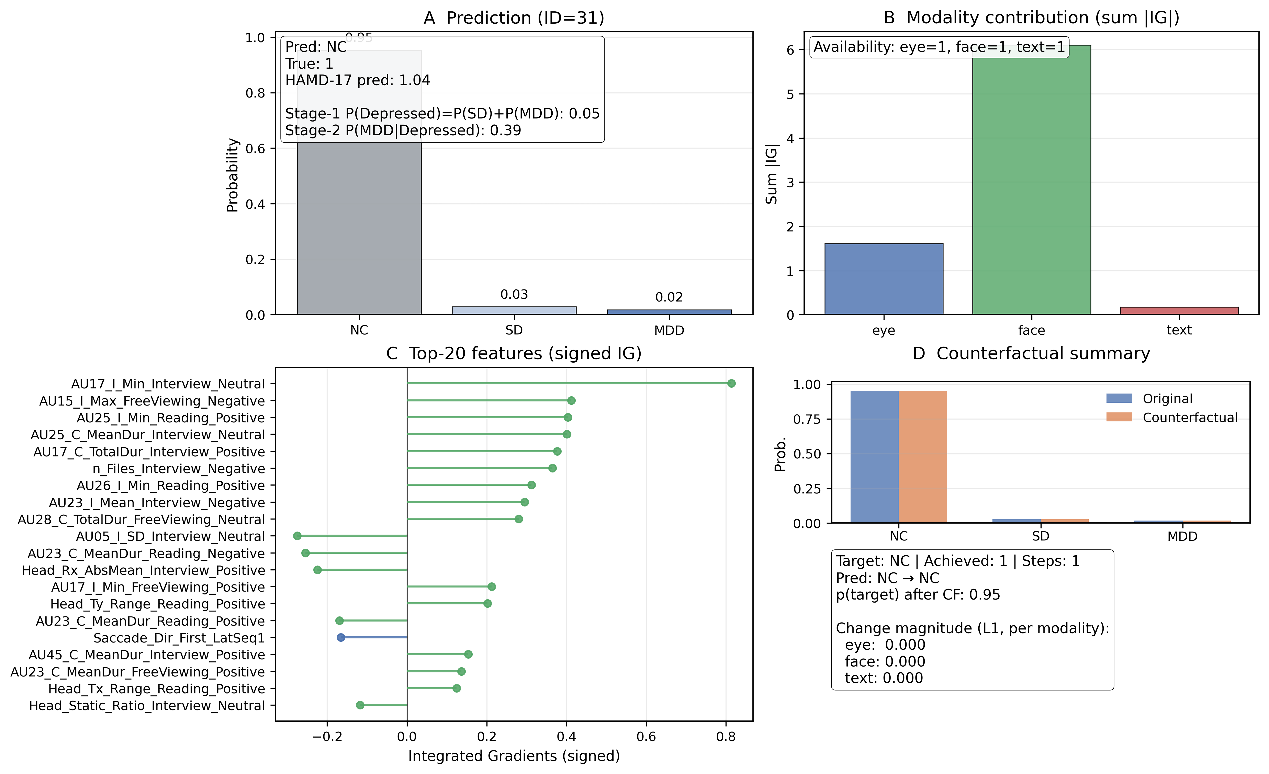 |
| --- |

**Supplementary Figure S11. Individual-level interpretability for a representative normal control case**

Legend. This figure presents the case-level interpretation generated by Baseline-3+ Route B for a representative normal control case. The panels summarize class probabilities, predicted HAMD-17 score, modality-level attribution magnitude, the leading Integrated Gradients features supporting the predicted class, and counterfactual analysis toward the NC target. The pattern is consistent with a stable low-risk profile, characterized by high NC probability, a very low predicted HAMD-17 score, and minimal perturbation required to preserve the NC classification. Abbreviations: HAMD-17, 17-item Hamilton Depression Rating Scale; IG, Integrated Gradients; MDD, major depressive disorder; NC, normal control; SD, subthreshold depression.

| 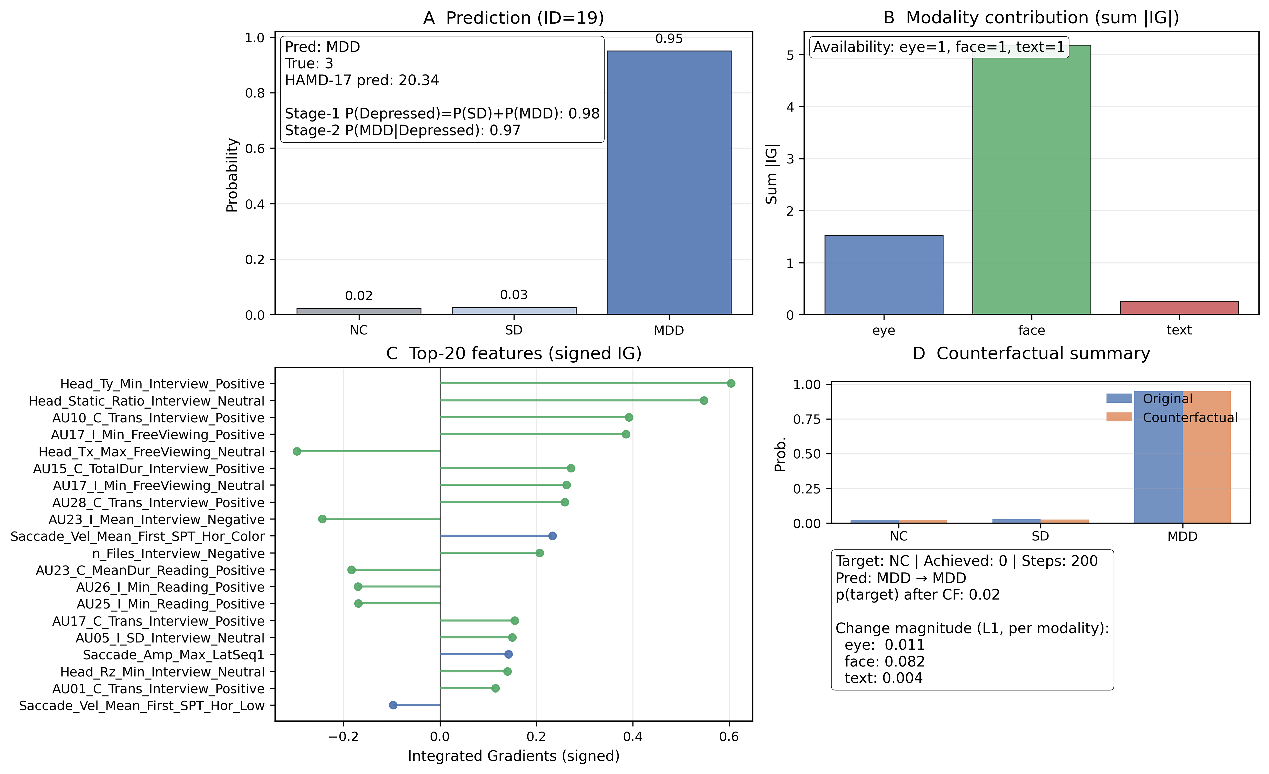 |
| --- |

**Supplementary Figure S12. Individual-level interpretability for a representative major depressive disorder case**

Legend. This figure presents the case-level interpretation generated by Baseline-3+ Route B for a representative major depressive disorder case. The panels summarize class probabilities, predicted HAMD-17 score, modality-level attribution magnitude, the leading Integrated Gradients features supporting the predicted class, and counterfactual analysis toward the NC target. The pattern is consistent with a stable high-risk profile, characterized by high probability at both stages of the hierarchical route, a high predicted HAMD-17 score, and failure of counterfactual conversion to NC within the perturbation limit. Abbreviations: HAMD-17, 17-item Hamilton Depression Rating Scale; IG, Integrated Gradients; MDD, major depressive disorder; NC, normal control; SD, subthreshold depression.
